# Protective effect of increased *O*-GlcNAc cycling against 6-OHDA induced Parkinson’s disease pathology

**DOI:** 10.1038/s41419-024-06670-1

**Published:** 2024-04-23

**Authors:** Dong Yeol Kim, Sang-Min Kim, Eun-Jeong Cho, Hyo-Bum Kwak, Inn-Oc Han

**Affiliations:** 1 Department of Biomedical Science, Program in Biomedical Science and Engineering, Inha University, Incheon, Korea; 2 Department of Kinesiology, Inha University, Incheon, Korea; 3 Department of Physiology and Biophysics, College of Medicine, Inha University, Incheon, Korea

**Keywords:** Parkinson's disease, Cell death in the nervous system, Parkinson's disease

## Abstract

This study aimed to elucidate the role of *O*-GlcNAc cycling in 6-hydroxydopamine (6-OHDA)-induced Parkinson’s disease (PD)-like neurodegeneration and the underlying mechanisms. We observed dose-dependent downregulation of *O*-GlcNAcylation, accompanied by an increase in *O*-GlcNAcase following 6-OHDA treatment in both mouse brain and Neuro2a cells. Interestingly, elevating *O*-GlcNAcylation through glucosamine (GlcN) injection provided protection against PD pathogenesis induced by 6-OHDA. At the behavioral level, GlcN mitigated motor deficits induced by 6-OHDA, as determined using the pole, cylinder, and apomorphine rotation tests. Furthermore, GlcN attenuated 6-OHDA-induced neuroinflammation and mitochondrial dysfunction. Notably, augmented O-GlcNAcylation, achieved through *O*-GlcNAc transferase (OGT) overexpression in mouse brain, conferred protection against 6-OHDA-induced PD pathology, encompassing neuronal cell death, motor deficits, neuroinflammation, and mitochondrial dysfunction. These collective findings suggest that *O*-GlcNAcylation plays a crucial role in the normal functioning of dopamine neurons. Moreover, enhancing *O*-GlcNAcylation through genetic and pharmacological means could effectively ameliorate neurodegeneration and motor impairment in an animal model of PD. These results propose a potential strategy for safeguarding against the deterioration of dopamine neurons implicated in PD pathogenesis.

## Introduction

Parkinson’s disease (PD) is a progressive neurodegenerative condition characterized by gradual reduction of dopamine levels in the striatum (ST), which is attributed to cumulative degeneration of dopaminergic (DA) neurons located in the substantia nigra (SN) pars compacta region of the midbrain [1]. Interactions of pathogenic factors, such as elevated oxidative stress, neuroinflammation, mitochondrial dysfunction, and apoptosis, collectively contribute to neurodegeneration in PD [2]. At present, the marketed treatments for PD are primarily focused on alleviating symptoms through dopaminergic medications, with no therapies currently available that can effectively target the underlying neurodegenerative process [3].

Among the various models of PD, the 6-hydroxydopamine (6-OHDA) rodent model has received significant attention for its contribution to advancing our understanding of PD pathology and the identification of novel therapeutic approaches [4]. 6-OHDA is a highly selective neurotoxin that induces degeneration in the nigrostriatal pathway [5]. One prominent feature of this toxin is its capacity to induce detectable motor impairments mirroring certain aspects of the motor symptoms of PD, such as bradykinesia [6, 7]. Moreover, rodents exposed to 6-OHDA display a rotational behavioral response following subcutaneous administration of apomorphine on the side opposite the lesioned hemisphere [8], which allows for assessment of the extent of the lesion. A significant number of animals with denervation induced by 6-OHDA, when exposed to chronic levodopa treatment, exhibit involuntary movements resembling levodopa-induced dyskinesia observed in patients [9, 10]. 6-OHDA inhibits mitochondrial complexes I and IV in the electron transport chain, leading to the generation of reactive oxygen species within neurons [11], and induces neuroinflammation that triggers apoptosis of nigrostriatal neuron [12].

Studies have revealed mitochondrial dysfunction primarily stemming from reduced mitochondrial complex I activity in PD [5, 13]. Mitochondria play essential roles in cellular function and homeostasis, underscoring the importance of maintaining mitochondrial quality. One mechanism through which mitochondria ensure a proper network and population is through balancing the processes of fission and fusion [14]. These fusion-fission cycles are crucial for preserving a healthy mitochondrial population by ensuring that functional mitochondria continuously undergo exchange and mixing of their contents [15]. SN neurons have particularly long axons, highlighting the crucial role of mitochondrial transport and anchoring in providing energy for the numerous docking events occurring within the synaptic microenvironment [16]. Consequently, mitochondrial fragmentation is a distinctive feature of PD brain [17].

Substantial evidence indicates a strong link between enhanced inflammatory response and PD. An earlier study showed the presence of activated microglia in the SN of postmortem PD cases [18]. Numerous subsequent investigations have documented increased microglial activation and elevated levels of pro-inflammatory cytokines in brain tissues and cerebrospinal fluid of individuals with PD, supporting this association [19, 20]. Moreover, cytokines, such as interleukin‐1β (IL‐1β) and tumor necrosis factor-alpha (TNF-α), are reported to contribute to neuronal damage and neurodegeneration, signifying a key role in the development of PD [21, 22].

*O*-GlcNAcylation is an intracellular post-translational modification that occurs on serine and threonine residues of proteins. This modification is remarkably dynamic, involving the addition of N-acetylglucosamine, a single monosaccharide unit. Attachment of *O*-GlcNAc to proteins is catalyzed by the enzyme *O*-GlcNAc transferase (OGT) while its removal is facilitated by *O*-GlcNAcase (OGA) [23]. Extensive research has explored the impact of *O*-GlcNAcylation in the context of neurodegenerative diseases. Although the results obtained to date are controversial, a substantial body of research consistently suggests that increased *O*-GlcNAcylation offers protection against neurodegeneration. A progressive decline in *O*-GlcNAc levels in human brain occurs during the course of normal aging and Alzheimer’s disease (AD) [24]. Additionally, earlier studies have shown that pharmacological elevation of *O*-GlcNAc levels could effectively alleviate memory impairment in AD mouse models, potentially through reducing protein aggregation [25, 26]. Relative to the extensive research focus on AD cases, the involvement of *O*-GlcNAcylation in the initiation and progression of PD has garnered comparatively less attention. Moreover, the majority of research examining the significance of *O*-GlcNAcylation in PD has primarily concentrated on the impact of *O*-GlcNAc on aggregation of α-synuclein that is genetically and neuropathologically linked to PD. Data from both in vivo and in vitro studies have shown a significant association of elevated *O*-GlcNAc levels with the capacity to impede aggregation of α-synuclein [27–29]. Conversely, other reports suggest that elevated *O*-GlcNAcylation promotes α-synuclein aggregation [30]. Given that aggregation of α-synuclein is a highly complex process, the effects of *O*-GlcNAcylation on α-synuclein levels and toxicity may vary depending on site-specific modifications. Irrespective of its impact on α-synuclein, it is plausible to postulate that *O*-GlcNAcylation intricately intersects with the degenerative pathways activated during progression of PD. However, to date, there has been a paucity of research attention on the functional implications of *O*-GlcNAc cycling in the context of neuronal damage associated with PD, particularly its relationship with neuroinflammation and mitochondrial dysfunction.

In this study, we provide evidence that changes in *O*-GlcNAc play a pivotal role in the degeneration of dopamine neurons. The finding that enhancement of *O*-GlcNAcylation not only mitigates motor deficits but also ameliorates neuroinflammation and mitochondrial dysfunction associated with the condition holds significant implications for the development of novel interventions for PD. The neuroprotective effect of elevated *O*-GlcNAcylation highlights its potential utility as a key regulator of neuronal survival and functionality in the context of PD.

## Result

### 6-OHDA regulates O-GlcNAc cycling in Neuro2a cells and mouse brain

**Fig. 1:**
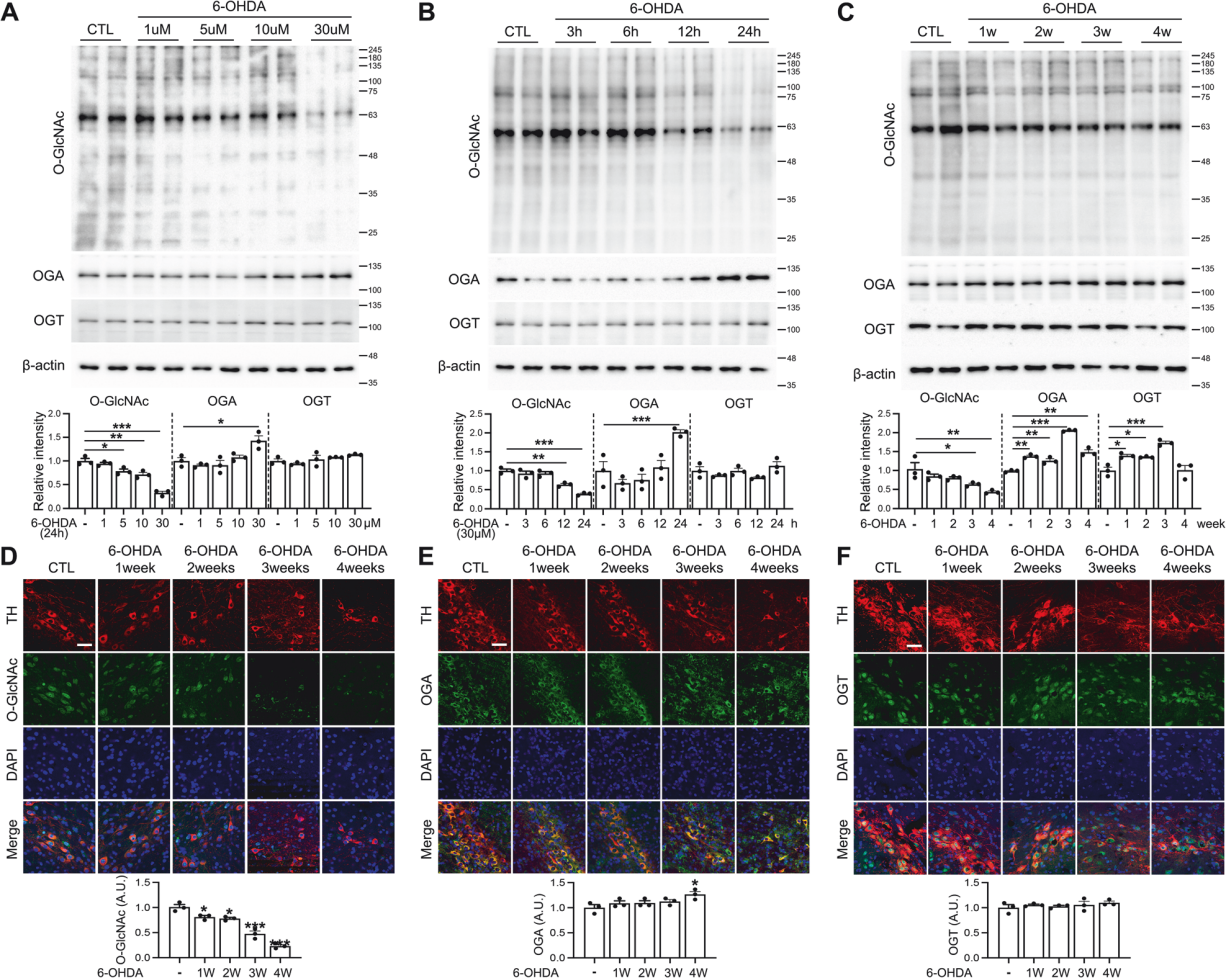
Alterations in *O*-GlcNAc cycling in Neuro2a cells and mouse brain in response to 6-OHDA. **A** Neuro2a cells were exposed to 6-OHDA at concentrations of 1, 5, 10, and 30 μM for 24 h. Whole-cell lysates were prepared and analyzed via immunoblot using antibodies specific for *O*-GlcNAc, OGA, OGT, and β-actin. The graphs depict densitometric quantification of *O*-GlcNAc, OGA, and OGT levels normalized to β-actin (*n* = 3/group). **B** Neuro2a cells were treated with 30 μM 6-OHDA for 3, 6, 12, and 24 h. Whole-cell lysates were used for immunoblot analysis with antibodies against *O*-GlcNAc, OGA, OGT, and β-actin. The graphs represent densitometric quantification of *O*-GlcNAc, OGA, and OGT, normalized to β-actin (*n* = 3/group). **C**–**F** Mice received injections of 3 μg 6-OHDA into the right striatum of the brain and were sacrificed after 1, 2, 3, and 4 weeks. **C** Representative western blot images of *O*-GlcNAc, OGA, OGT, and β-actin from the SN region of mouse brain are displayed and the graphs illustrate densitometric quantification of *O*-GlcNAc, OGA, and OGT normalized to β-actin (*n* = 3/group). **D** Representative immunofluorescence images (40X) of *O*-GlcNAc, TH, DAPI, and merged images in the SN region of mouse brain at 4 weeks (*n* = 3/group). **E** Representative immunofluorescence images (40X) of OGA, TH, DAPI, and merged images in the SN region of mouse brain at 4 weeks (*n* = 3/group). **F** Representative immunofluorescence images (40X) of OGT, TH, DAPI, and merged images in the SN region of mouse brain at 4 weeks (*n* = 3/group). The scale bar represents 50 μm. Data are presented as mean ± SEM; ^*^p < 0.05, ^**^p < 0.01, ^***^p < 0.001 vs control group. Statistical analysis was performed using one-way analysis of variance (ANOVA) with Tukey’s post hoc multiple comparison test.

To investigate the involvement of *O*-GlcNAc cycling in progression of PD, we treated Neuro2a cells with 6-OHDA, a well-known neurotoxin that induces PD-like symptoms, and analyzed the changes in *O*-GlcNAcylation. Initially, Neuro2a cells were exposed to various concentrations of 6-OHDA and *O*-GlcNAc alterations observed over a 24 h time-course via immunofluorescence (IF) staining. Notably, 6-OHDA elicited a significant reduction in *O*-GlcNAc levels in a time- and concentration-dependent manner (Suppl. Fig. 1A, B). Subsequently, western blot analysis was conducted to examine changes in *O*-GlcNAcylation and expression of key enzymes, OGT and OGA, in Neuro2a cells. 6-OHDA induced a time and concentration-dependent reduction in *O*-GlcNAcylation. While OGT expression remained relatively unchanged, we observed a significant increase in OGA expression at 24 h and at the highest concentration (30 μM) of 6-OHDA (Fig. 1A, B). We further investigated the changes in *O*-GlcNAcylation induced by 6-OHDA in vivo using a mouse model. Following injection of 6-OHDA into the right ST of mouse brain, we examined *O*-GlcNAc alterations and levels of OGT and OGA weekly over a period of 4 weeks via western blot and IF analyses. The collective results consistently showed a decrease in *O*-GlcNAcylation in the SN region from the first week, which progressively continued until week 4 (Fig. 1C, D). Interestingly, OGA and OGT levels remained relatively unchanged over the 4 weeks according to the IF results, while western blot results revealed an increase in both OGT and OGA, starting from week 1 (Fig. 1E, F).

### GlcN exerts a protective effect on functional and molecular degeneration induced by 6-OHDA

**Fig. 2:**
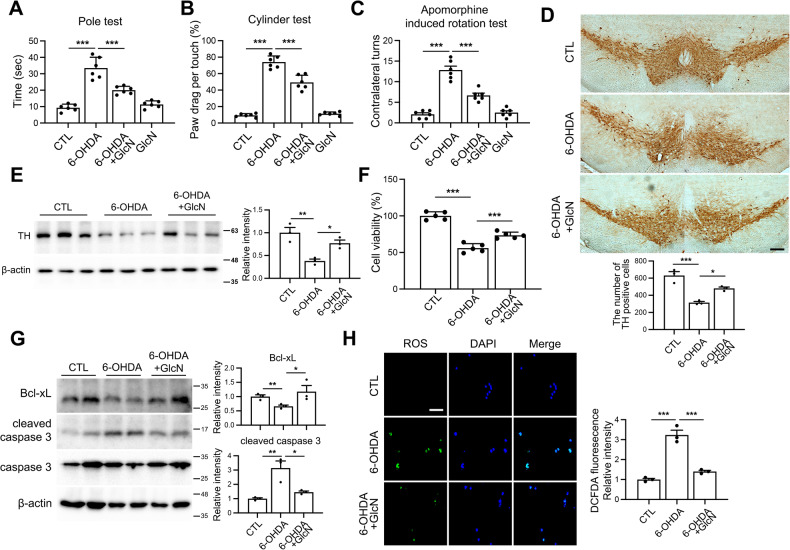
Protective effects of GlcN against PD pathogenesis in a 6-OHDA-induced PD Model. 6-OHDA was injected into the right striatum of mouse brains. Some groups received intraperitoneal injections of GlcN at a concentration of 20 mg/kg three times a week for 4 weeks. **A**–**C** Behavioral tests, including the pole test (**A**), cylinder test (**B**), and apomorphine rotation test (**C**), were conducted 4 weeks after the injections (*n* = 6/group). **D** Dopaminergic neurons in the SN of mouse brains were visualized using immunohistochemistry with TH antibodies (*n* = 3/group). The scale bar represents 100 μm. **E** Western blot analysis of SN tissue lysates was performed using TH and β-actin antibodies. TH quantification was normalized to β-actin (*n* = 3/group). **F** Cell viability in Neuro2a cells was assessed after treatment with 6-OHDA (30 μM) and GlcN (1 mM) for 24 h (*n* = 5/group). **G** Representative Western blot analysis of Neuro2a cells treated with 6-OHDA (30 μM) and GlcN (1 mM) for 24 h using BcL-xL, cleaved caspase 3, caspase 3, and β-actin antibodies. BcL-xL quantification was normalized to β-actin, while cleaved caspase 3 quantification was normalized to caspase 3 (*n* = 3/group). **H** Representative detection of reactive oxygen species in Neuro2a cells after 24 h of treatment with 6-OHDA (30 μM) and GlcN (1 mM) using DCFDA fluorescence (green) and DAPI (blue) staining (*n* = 3/group). Data are presented as mean ± SEM; ^*^p < 0.05, ^**^p < 0.01, ^***^p < 0.001. Statistical analysis was performed using one-way ANOVA with Tukey’s post hoc multiple comparison test.

To investigate the functional impact of suppression of *O*-GlcNAcylation by 6-OHDA in PD pathogenesis, mice were treated with glucosamine (GlcN), to increase *O*-GlcNAcylation. GlcN was administered intraperitoneally three times a week for 4 weeks and its effects on motor impairments induced by 6-OHDA initially validated through behavioral tests. In the pole, cylinder, and apomorphine-induced rotational tests, 4-week 6-OHDA treatment led to PD-specific motor deficits, as indicated by the increased latency to descend, high percentage of paw dragging, and contralateral turns compared to the control group (Fig. 2A, B, C). All behavioral deficits were reversed following GlcN treatment. Next, immunohistochemical staining was conducted to assess the effects of GlcN on degeneration of dopaminergic neurons in the SN region induced by 6-OHDA. As shown in Fig. 2D, mice injected with 6-OHDA displayed a significant reduction in the number of TH-positive neurons in the SN region compared to their control counterparts, which was effectively ameliorated by GlcN (Fig. 2D). Similarly, western blot analysis showed a decrease in TH levels induced by 6-OHDA, which was subsequently restored by GlcN (Fig. 2E). Moreover, the 6-OHDA-induced decrease in viability of Neuro2a cells was reversed by GlcN (Fig. 2F). Consistent results were obtained upon analysis of proapoptosis markers. Treatment of Neuro2a cells with 6-OHDA resulted in a decrease in Bcl-xL and increase in cleaved caspase 3 levels, indicative of enhanced apoptosis, which were restored to normal levels in the presence of GlcN (Fig. 2G). Furthermore, 6-OHDA induced elevation of ROS levels, which were subsequently reduced upon treatment with GlcN (Fig. 2H).

### 6-OHDA-induced O-GlcNAcylation changes are reversed by GlcN treatment

**Fig. 3:**
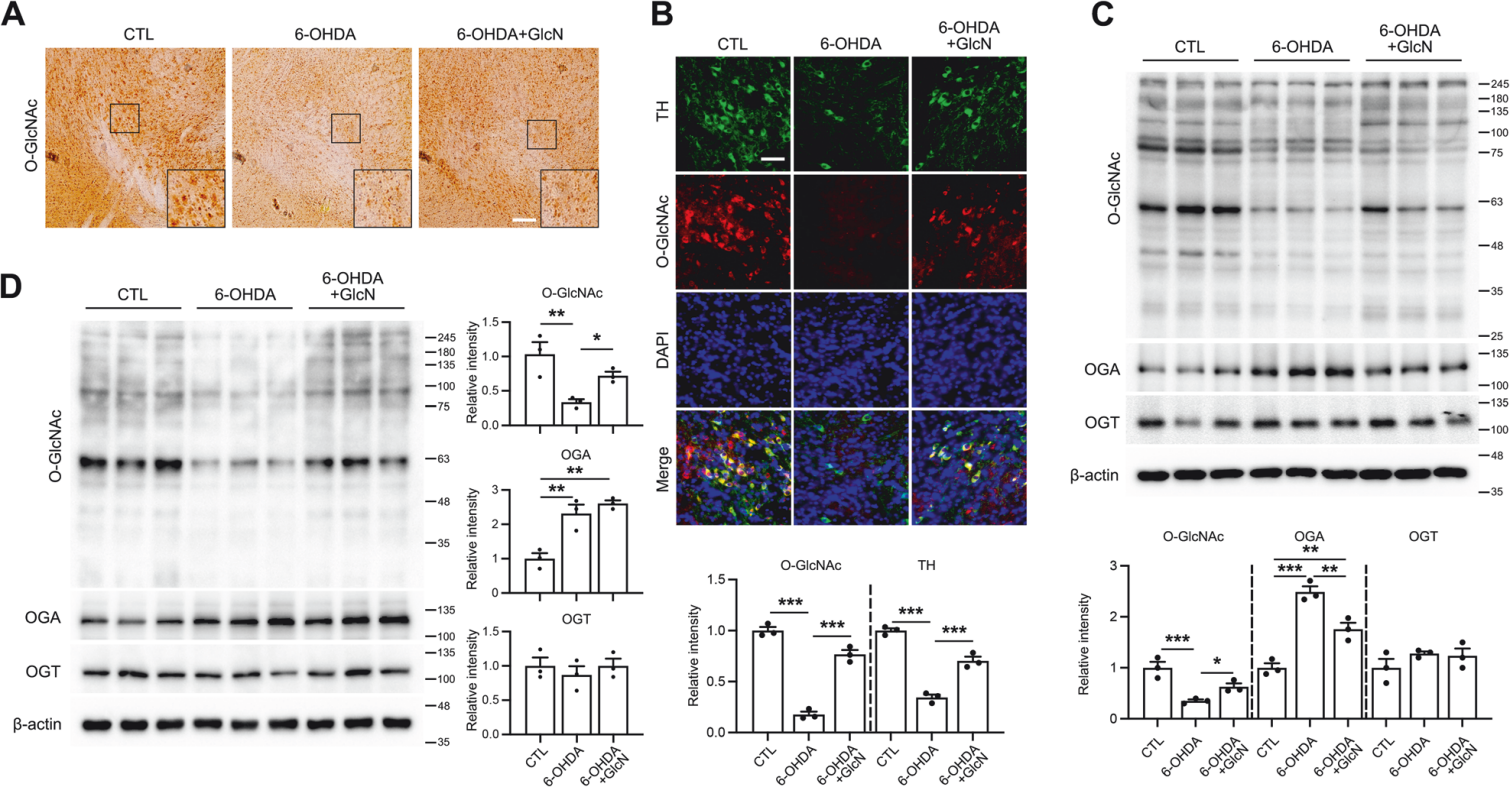
Effects of GlcN on *O*-GlcNAc cycling in the 6-OHDA-induced PD model. 6-OHDA (3 μg) was injected into the right striatum of mouse brains, and in some groups, GlcN was administered at a concentration of 20 mg/kg through intraperitoneal injection three times a week for 4 weeks. **A** Representative immunohistochemical staining (40X) of *O*-GlcNAc in the SN of mouse brains 4 weeks after treatment. Scale bar represents 100 μm (*n* = 3/group). **B** Representative immunofluorescent staining (40X) showing *O*-GlcNAc (red), TH (green), DAPI (blue) and merged images in the SN of mouse brains. Scale bar represents 50 μm. (*n* = 3/group). **C** Western blot of *O*-GlcNAc, OGA, OGT, and β-actin in tissue extracts from the SN of mouse brains. The quantification of *O*-GlcNAc, OGA, and OGT was normalized to β-actin (*n* = 3/group). **D** Neuro2a cells were treated with 6-OHDA (30 μM) with or without GlcN (1 mM) for 24 h. Western blot analysis of *O*-GlcNAc, OGA, OGT, and β-actin are showing. The quantification of *O*-GlcNAc, OGA, and OGT was normalized to β-actin (*n* = 3/group). Data are presented as mean ± SEM; ^*^p < 0.05, ^**^p < 0.01, ^***^p < 0.001. Statistical analysis was conducted using one-way ANOVA with Tukey’s post hoc multiple comparison test.

Next, we examined whether GlcN regulates the diminished *O*-GlcNAc cycling induced by 6-OHDA, both in vivo and in vitro. In Neuro2a cells, GlcN restored *O*-GlcNAcylation suppressed by 6-OHDA (Suppl. Fig. 2). Consistent with in vitro findings, immunostaining of the SN region in our mouse model showed a significant increase in overall *O*-GlcNAcylation following treatment with GlcN plus 6-OHDA compared to mice treated only with 6-OHDA (Fig. 3A). Furthermore, IF staining confirmed that GlcN restored *O*-GlcNAcylation that was decreased by 6-OHDA-induced (Fig. 3B). Western blot analysis yielded consistent results. In both the mouse model (Fig. 3C) and Neuro2A cells (Figs. 3D), 6-OHDA induced a reduction in *O*-GlcNAcylation, which was subsequently restored by GlcN. Interestingly, OGA exhibited a complex regulation pattern. While the increase in 6-OHDA-induced OGA was suppressed by GlcN in Neuro2a cells, 6-OHDA-induced elevation of OGA in the brain was further heightened by GlcN in the mouse model (Fig. 3C, D). In contrast, the OGT level was not significantly altered in response to 6-OHDA, with or without GlcN.

### GlcN protects against 6-OHDA-induced mitochondrial dysfunction

**Fig. 4:**
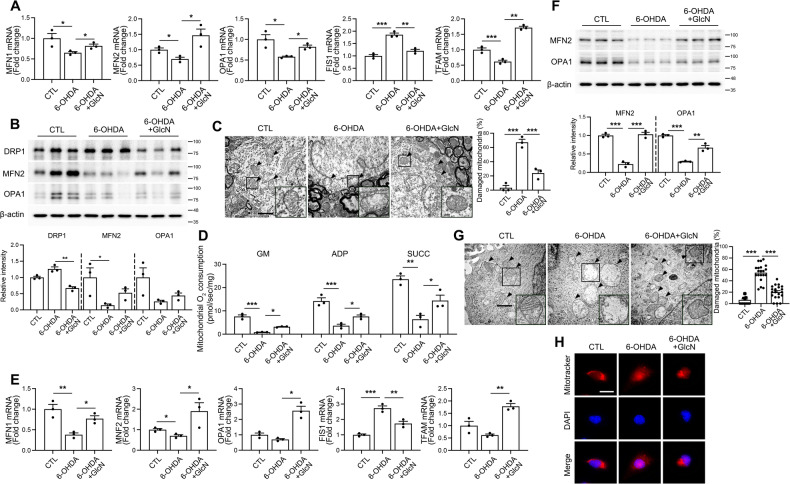
Regulation of mitochondrial dynamics and stability by GlcN in 6-OHDA-induced mice. **A**–**E** 3 μg of 6-OHDA was injected into the right striatum of mouse brains, and in some groups, GlcN (20 mg/kg) was intraperitoneally administered three times a week for 4 weeks. **A** RNA was extracted from the SN in mouse brains, and real-time PCR was conducted using primers for MFN1, MFN2, OPA1, FIS1, and TFAM to assess changes in mRNA expression. **B** Western blot analysis of SN tissue extracts using DRP1, MFN2, OPA1, and β-actin antibodies. Quantification of DRP1 and MFN2 was normalized to β-actin (*n* = 3/group). **C** Mitochondrial morphology changes in the SN were examined using electron microscopy, with subsequent plotting of the percentage of damaged mitochondria (*n* = 3/group). Scale bar represents 2 μm. **D**–**H** Neuro2a cells were treated with 6-OHDA (30 μM) with or without GlcN (1 mM) for 24 h. **D** Mitochondrial oxygen consumption was measured after treatment with glutamate/malate, ADP, and succinate (*n* = 3/group). **E** RNA was extracted from the cells, and real-time PCR was conducted using primers for MFN1, MFN2, OPA1, FIS1, and TFAM to assess changes in mRNA expression (*n* = 3/group). **F** Western blot analysis was performed on cell lysates using MFN2, OPA1, and β-actin antibodies. Representative blots are shown and quantification of MFN2 and OPA1 was normalized to β-actin (*n* = 3/group). **G** Mitochondrial morphology changes in Neuro2a cells were examined using electron microscopy, and the proportion of damaged mitochondria were graphically represented (*n* = 20/group). Scale bar represents 2000 nm. **H** Mitochondria were visualized using Mitotracker fluorescent staining. Scale bar represents 50 μM. Data are presented as mean ± SEM; ^*^p < 0.05, ^**^p < 0.01, ^***^p < 0.001. Statistical analysis was performed using one-way ANOVA with Tukey’s post hoc multiple comparison test.

Mitochondria are highly dynamic organelles that undergo regulated fission-fusion processes. Mitochondrial stability plays a critical role in controlling the progression of PD [31]. Accordingly, we investigated the impact of 6-OHDA on mitochondrial dynamics in the presence and absence of GlcN. The mRNA levels of mitochondrial fusion markers, including MFN1, MFN2, and OPA1, in brains of mice were reduced by 6-OHDA and significantly increased following GlcN treatment (Fig. 4A). Conversely, the mitochondrial fission marker, FIS1, elevated by 6-OHDA, was decreased in the presence of GlcN (Fig. 4A). Consistent with mRNA results, protein levels of the mitochondrial fusion markers, MFN2 and OPA, were reduced by 6-OHDA and restored by GlcN (Fig. 4B). Furthermore, DRP1, a fission marker upregulated by 6-OHDA, was attenuated in the presence of GlcN (Fig. 4B). We further assessed mitochondrial integrity in brains of mice treated with 6-OHDA using transmission electron microscopy. 6-OHDA treatment resulted in a distinct pattern of mitochondrial damage, which was significantly restored by GlcN (Fig. 4C). We additionally validated the functional and morphological changes of mitochondria through experiments using Neuro2a cells. Mitochondrial oxygen consumption was measured to assess mitochondrial function. Mitochondrial activity and oxygen consumption, which had been reduced in brains of 6-OHDA-treated mice, were restored following treatment with GlcN (Fig. 4D). Furthermore, 6-OHDA induced a decrease in mitochondrial fusion markers and increase in fission markers (Fig. 4E, F), along with disruption of mitochondrial morphology (Fig. 4G). The collective changes were prevented by GlcN treatment. Moreover, fluorescent staining with MitoTracker revealed that 6-OHDA induced a significant reduction in the distribution of mitochondria, which was effectively restored following GlcN treatment (Fig. 4H).

### GlcN suppresses 6-OHDA-induced neuroinflammation

**Fig. 5:**
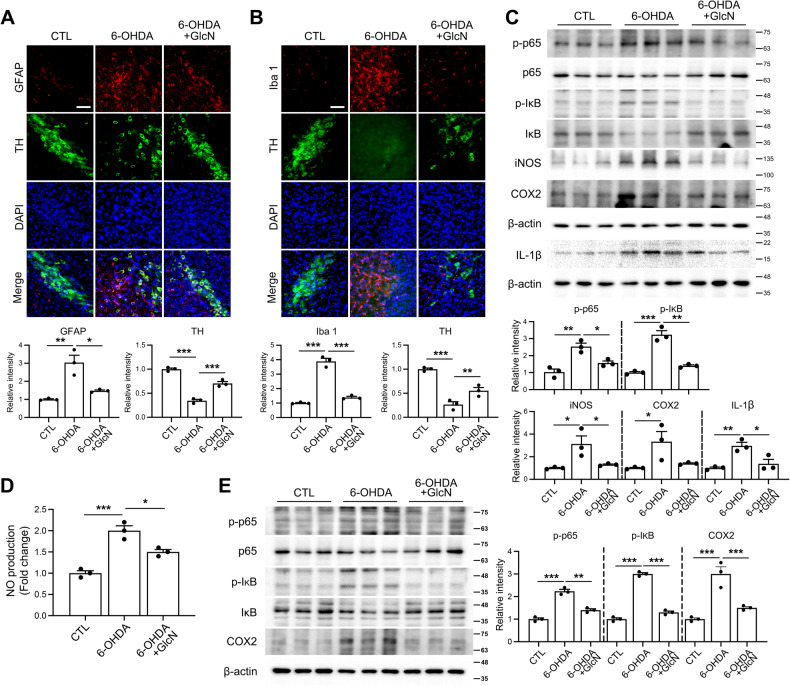
Effect of GlcN on 6-OHDA-induced neuroinflammation. **A**–**C** 3 μg of 6-OHDA was injected into the right striatum of mouse brains, and in some groups, GlcN was intraperitoneally administered at a concentration of 20 mg/kg three times a week for 4 weeks. **A** Representative immunofluorescent images for GFAP (red), TH (green), DAPI (blue), and a merged image of SN in mouse brains. Scale bar represents 50 μm. (*n* = 3/group). **B** Representative immunofluorescent staining in the SN of mouse brains, showing Iba1 (red), TH (green), DAPI (blue), and merged images. Scale bar represents 50 μm. (*n* = 3/group). **C** Western blot analysis of the SN tissue extracts in mouse brains using p-p65, p65, p-IκB, IκB, iNOS, COX2, IL-1β and β-actin antibodies. The quantification of p-p65 was normalized to p65, p-IκB was normalized to IκB, and the quantification of iNOS, COX2 and IL-1β was normalized to β-actin (*n* = 3/group). **D** and (**E**) Neuro2a cells after 24 h of treatment with 6-OHDA (30 μM) with or without GlcN (1 mM). Nitrite production was measured (**D**). Western blot analysis from cell lysates was performed using p-p65, p65, p-IκB, IκB, iNOS, COX2, and β-actin antibodies (**E**). The quantification of p-p65 was normalized to p65, p-IκB was normalized to IκB, and the quantification of iNOS and COX2 was normalized to β-actin (*n* = 3/group). Data are presented as mean ± SEM; ^*^p < 0.05, ^**^p < 0.01, ^***^p < 0.001. Statistical analysis was performed using one-way ANOVA with Tukey’s post hoc multiple comparison test.

We explored the regulatory effects of GlcN on glial activation in the brain triggered by 6-OHDA. In our experiments, 6-OHDA promoted astrocyte activation, as evident from elevated GFAP levels (Fig. 5A). Furthermore, we observed an increase in the number of activated microglia in brains of mice injected with 6-OHDA (Fig. 5B). Notably, both GFAP levels and activated microglia were significantly reduced following GlcN injection (Fig. 5A, B). Western blot analysis of inflammatory markers showed elevated levels of iNOS, COX2 and IL-1β, along with increased phosphorylation of the p65 subunit of NF-κB and IκB in brains of mice injected with 6-OHDA. GlcN injection significantly mitigated these inflammatory responses (Fig. 5C). These findings were further validated in Neuro2a cells. NO production, measured based on nitrite, increased in response to 6-OHDA treatment, and was effectively reduced upon co-treatment with GlcN (Fig. 5D). Additionally, pro-inflammatory signals, including phosphorylation of p65 and IκB as well as expression of COX-2 protein, were elevated in 6-OHDA-treated Neuro2a cells. This increase was inhibited upon treatment with GlcN (Fig. 5E).

### Neuronal overexpression of OGT exerts a protective effect on dopaminergic neurons under conditions of pathogenesis of 6-OHDA-induced PD

**Fig. 6:**
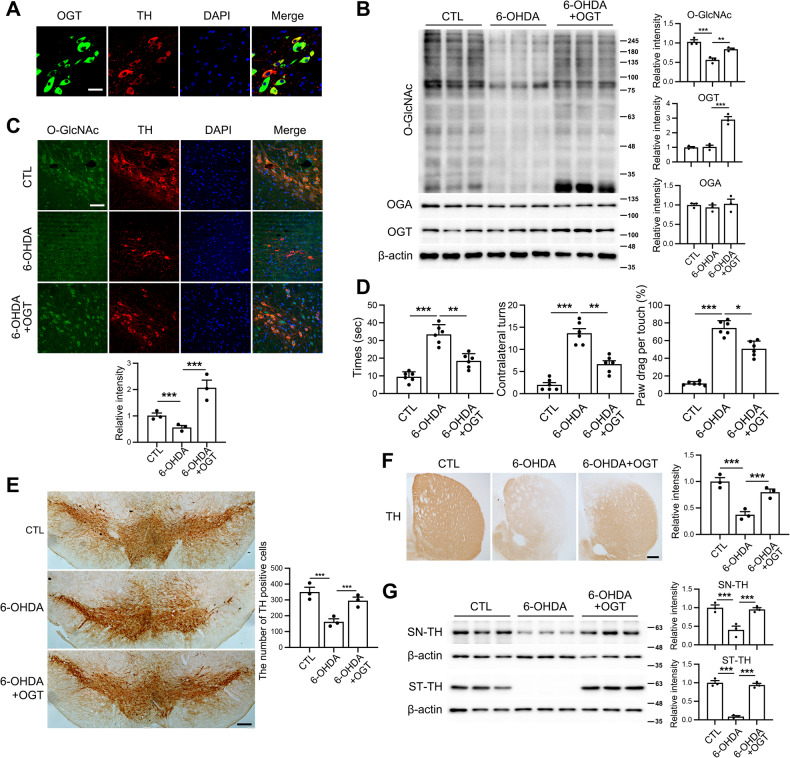
Effects of OGT overexpression on 6-OHDA-induced cytotoxicity in dopaminergic neuron in mouse. 3 μg of 6-OHDA was injected into the right striatum of mouse brains, followed by the injection of AAV-OGT overexpression virus (1 × 10^13^ genome copies per milliliter) into the SN region. The mice were sacrificed 4 weeks later. **A** Representative immunofluorescent staining of OGT and TH in the SN of mouse brains. Scale bar represents 50 μm. **B** Western blot analysis was performed on SN tissue extracts using *O*-GlcNAc, OGA, OGT, and β-actin antibodies. The quantification of *O*-GlcNAc, OGA, and OGT was normalized to β-actin (*n* = 3/group). **C** Representative immunofluorescent staining of *O*-GlcNAc (green), TH (red), DAPI, and merged images in dopaminergic neurons in the SN (*n* = 3/group). **D** Behavioral experiments were conducted using the pole test, cylinder test, and apomorphine rotation test 4 weeks after the injections (*n* = 6/group). **E** Dopaminergic neurons were visualized in the SN using immunohistochemistry with TH antibodies (*n* = 3/group). **F** Immunohistochemistry staining in the striatum was performed to confirm axon terminals of dopaminergic neurons using TH antibodies (*n* = 3/group). Representative stainings are shown. **G** Western blot analysis of the SN and striatum tissue extracts using TH and β-actin antibodies. The quantification of TH was normalized to β-actin (*n* = 3/group). Data are presented as mean ± SEM; ^*^p < 0.05, ^**^p < 0.01, ^***^p < 0.001. Statistical analysis was performed using one-way ANOVA with Tukey’s post hoc multiple comparison test.

To further elucidate the impact of *O*-GlcNAc cycling in the context of 6-OHDA-induced PD pathogenesis, we administered AAV-OGT virus into the ST region of mouse brains and assessed its effects on damage to TH neurons caused by 6-OHDA. Specifically, mice were subjected to viral injection following 6-OHDA treatment. After 4 weeks, OGT expression was observed in TH neurons in the AAV-OGT injected group (Fig. 6A). Our data showed that OGT overexpression significantly restored *O*-GlcNAcylation in the SN region (Fig. 6B) and TH neurons of SN (Fig. 6C) that had been suppressed by 6-OHDA. Western blot results revealed an increase in OGT protein while the OGA level remained unchanged in mouse brain following viral OGT overexpression (Fig. 6B). Analysis of behavioral performance showed that animals receiving OGT virus injections exhibited reversal of 6-OHDA-induced impairment of motor coordination and balance. This finding was validated by the OGT-overexpressing 6-OHDA group demonstrating a decrease in latency to descend in the pole test, contralateral turns in the rotation test, and paw drag per touch in the cylinder test compared to the 6-OHDA group (Fig. 6D). Furthermore, mice injected with 6-OHDA exhibited a reduced number of TH-positive neurons in both the ST and SN regions. Notably, OGT virus injection in 6-OHDA-treated mice induced a significant increase in the number of TH-positive neurons (Fig. 6E, F). Western blot analysis further confirmed significant reduction of TH protein levels in both the ST and SN regions, which were subsequently restored to levels comparable to those of the control group (Fig. 6G).

### OGT overexpression suppresses 6-OHDA-induced neuroinflammation and mitochondrial instability

**Fig. 7:**
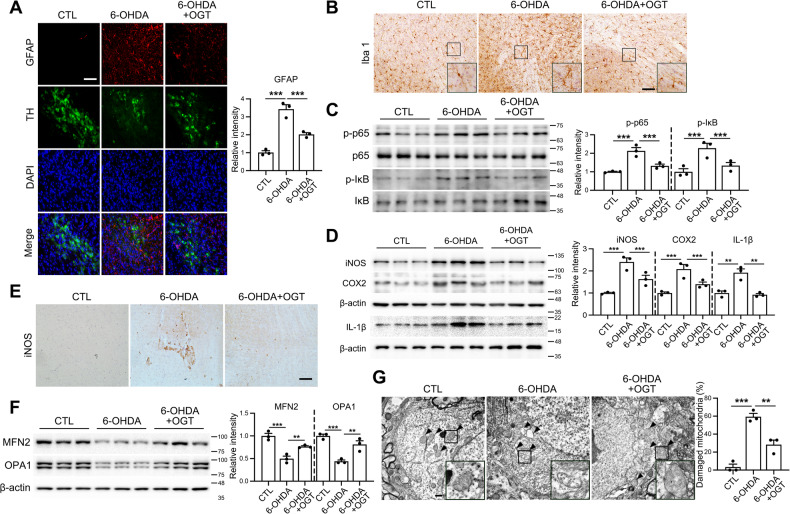
Effects of OGT overexpression on inflammatory response and mitochondrial stability induced by 6-OHDA in mouse. 3 μg of 6-OHDA was injected into the right striatum of mouse brains, and in some groups, AAV-OGT overexpression virus (1 × 10^13^ genome copies per milliliter) was injected into the SN region, after which the mice were sacrificed 4 weeks later. **A** Immunofluorescent staining was performed in the SN of mouse brains for GFAP (red), TH (green), DAPI (blue), and the merged image. Representative staining images are shown. Scale bar represents 50 μm. (*n* = 3/group). **B** Representative immunohistochemistry for Iba1 staining in the SN. Scale bar represents 100 μm. **C** Western blot analysis of the SN tissue using p-p65, p65, p-IκB, and IκB antibodies. Quantification of p-p65 was normalized to p65, and p-IκB was normalized to IκB. (*n* = 3/group). **D** Western blot analysis of iNOS, COX2 and IL-1β in SN tissue using β-actin antibodies. Quantification of iNOS, COX2 and IL-1β was normalized to β-actin. (*n* = 3/group). **E** Immunohistochemistry was employed to visualize iNOS in the substantia nigra. Representative images are shown. Scale bar represents 100 μm. **F** Western blot analysis of SN tissue lysate using MFN2, OPA1, and β-actin antibodies. Quantification of MFN2 and OPA1 was normalized to β-actin (*n* = 3/group). **G** Mitochondrial morphology changes in the SN were examined using electron microscopy, and the percentage of damaged mitochondria was depicted graphically (*n* = 3/group). Scale bar represents 2000 nm. Data is presented as mean ± SEM; ^**^p < 0.01, ^***^p < 0.001. Statistical analysis was performed using one-way ANOVA with Tukey’s post hoc multiple comparison test.

We further investigated the regulatory effects of OGT on the inflammatory response in 6-OHDA-treated brain. 6-OHDA led to an increase in astrocyte activation, as indicated by elevated levels of GFAP, which was effectively suppressed under conditions of OGT overexpression (Fig. 7A). Additionally, Iba1 staining revealed an increase in activated microglia in brains of 6-OHDA-injected mice, which was suppressed by OGT overexpression (Fig. 7B). Proinflammatory signaling was further examined via western blot analysis. The results showed increased phosphorylation of p65 and IκB in the 6-OHDA group, which was significantly reduced in the OGT overexpression group (Fig. 7C). Additionally, 6-OHDA-induced upregulation of of iNOS, COX-2 and IL-1β was markedly suppressed by OGT overexpression (Fig. 7D, E). Examination of mitochondrial stability demonstrated that the levels of mitochondrial fusion markers, MFN2 and OPA1, were decreased by 6-OHDA and restored by OGT (Fig. 7F). Similarly, 6-OHDA damaged mitochondrial integrity, which was restored following OGT overexpression (Fig. 7G).

## Discussion

In the current study, we provide evidence that *O-*GlcNAc cycling serves as a critical signal for regulating physiological function, survival, and degeneration of dopamine neurons in a 6-OHDA-induced mouse model of PD. Multiple conflicting results have been reported regarding the role of *O*-GlcNAcylation in the onset and progression of PD. However, previous studies on the impact of *O*-GlcNAcylation on PD have mainly focused on *O*-GlcNAc modification of α-synuclein. In the current investigation, we employed a 6-OHDA-induced PD model with no α-synuclein accumulation [32], to examine whether *O*-GlcNAcylation is implicated in functional and pathological alterations in DA neurons through pathways independent of α-synuclein. In our experiments, 6-OHDA reduced *O*-GlcNAc levels in both Neuro2a cells and mouse brain. Interestingly, although 6-OHDA induced a decrease in overall *O*-GlcNAc, both OGA and OGT levels were increased in response to 6-OHDA in mouse brain. Previous examinations have suggested that cells coordinate the expression of OGT and OGA to prevent drastic fluctuations in *O*-GlcNAcylation levels, and the expression of OGT and OGA is responsive to changes in cellular *O*-GlcNAc levels. For example, multiple studies have demonstrated a decrease in OGT protein levels under conditions of pharmacological inhibition of OGA in various cell lines [33–35]. Conversely, OGA protein levels are downregulated in response to OGT inhibition, knockdown, or knockout in a number of cell lines [36–38]. These findings suggest that the relationship between the opposing enzymes, OGT and OGA, may not consistently follow an inverse pattern in response to changes in *O*-GlcNAcylation, thus raising an intriguing question about the nuanced and complex regulatory mechanisms governing *O*-GlcNAc dynamics, which warrant further exploration.

Our collective data clearly demonstrate that upregulation of *O*-GlcNAcylation through GlcN supplementation or virus-mediated OGT overexpression rescues both functional and pathological PD phenotypes induced by 6-OHDA, supporting the emerging concept that *O*-GlcNAcylation plays a beneficial role in promoting cell survival and protecting neurons from various cellular stresses [39]. One of the primary targets of altered *O*-GlcNAc cycling triggered by cellular stress is potentially mitochondria [40]. SN neurons have particularly long axons, and thus mitochondrial trafficking and anchoring are essential to provide energy for the numerous docking events occurring in the synaptic microenvironment [16]. A role of mitochondrial dysfunction in the pathogenesis of PD has long been considered. Accumulating evidence suggests that mitochondrial dysfunction and impaired mitophagy play a significant role in PD development [41, 42]. Recently, *O*-GlcNAcylation has emerged as a pivotal regulator of mitochondrial function [43, 44]. Our findings provide novel evidence that a decrease in *O*-GlcNAcylation plays a critical role in mitochondrial dysfunction, contributing to the pathogenesis of PD. Consistent results from in vitro and in vivo studies indicated that 6-OHDA treatment triggers mitochondrial dysfunction characterized by reduced fusion, increased fission, decreased O_2_ consumption, and compromised mitochondrial integrity. These effects could be ameliorated by GlcN treatment or OGT overexpression. In keeping with this finding, previous research has shown that GlcN treatment enhances mitochondrial respiration [45]. The precise mechanisms by which *O*-GlcNAc cycling regulates mitochondrial function remain to be resolved. It is plausible that 6-OHDA impacts mitochondrial function by modulating *O*-GlcNAcylation of mitochondrial proteins. However, comprehensive elucidation of *O*-GlcNAcylation of specific mitochondrial proteins in response to 6-OHDA and/or GlcN and their functions requires substantial research effort and extends beyond the scope of this study. The complexity of signaling pathways that affect mitochondrial integrity and function, along with the indirect effects of nutrient states on *O*-GlcNAcylation, adds an additional layer of intricacy to our understanding of the role of *O*-GlcNAc in mitochondrial integrity. Nonetheless, these findings offer valuable insights into the potential utility of pharmacological interventions targeting *O*-GlcNAc to mitigate mitochondrial dysfunction. Furthermore, the results pave the way for future research on the specific mitochondrial proteins affected by *O*-GlcNAcylation and their contributions to the intricate network of mitochondrial functions.

In the pathophysiology of PD, neuroinflammation characterized by pronounced microglial and astrocytic activation in the SN plays a pivotal role in disease progression [46–48]. Indeed, activation of microglia has been reported in animal models of PD induced by 6-OHDA [49]. The NF-κB signaling pathway is a key player in this process [50]. Ghosh and co-workers demonstrated that selective inhibition of NF-κB prevents the degeneration of DA neurons in the SN in a mouse model of PD [51]. Similarly, targeting the NF-κB pathway in murine PD models may halt progression of the disease [52]. In the current study, 6-OHDA induced not only the activation of microglia and astrocytes but also NF-κB signaling and induction of proinflammatory molecules, including iNOS, COX-2 and IL-1β. Importantly, both glial activation and inflammatory responses were mitigated by enhancing *O*-GlcNAcylation through GlcN supplementation and OGT overexpression in the SN. These findings are in alignment with our previous results, which indicate that reduced *O*-GlcNAcylation is associated with activation of microglia, NF-κB, and the induction of proinflammatory molecules following ischemic brain injury or bacterial lipopolysaccharide stimulation [53, 54]. We have additionally shown that activation of glial cells and neuroinflammation induced by hypoxic insult are associated with decreased *O*-GlcNAcylation and can be effectively suppressed by enhancing *O*-GlcNAcylation [55, 56]. Our consistent findings across multiple studies underscore the close association between reduced *O*-GlcNAcylation and neuroinflammation, highlighting the potential for modulating PD-related inflammation through *O*-GlcNAc cycling. Notably, since the SN is particularly vulnerable to neuroinflammation due to its high microglial density [57], regulation of neuroinflammation via *O*-GlcNAc cycling holds promise for future research in the development of PD therapeutics. However, the issue of whether *O*-GlcNAc cycling directly regulates neuroinflammation or modulates inflammation through the regulation of other neural cell activities remains unclear. Additionally, the specific targets of inflammatory signals regulated by *O*-GlcNAc cycling await further elucidation in future investigations.

In summary, our study demonstrates that elevation of *O*-GlcNAcylation in a mouse model of PD through GlcN supplementation or OGT overexpression has the potential to alleviate PD, possibly by reprogramming mitochondrial function and reducing neuroinflammation in the SN. Our findings open new avenues for future research, highlighting the importance of *O*-GlcNAcylation as a target for the development of neuroprotective strategies against PD. Nevertheless, several fundamental questions warrant further exploration, including the identification of the target proteins crucial for regulating mitochondrial function or inflammatory responses. Given that many *O*-GlcNAcylated proteins possess multiple *O*-GlcNAcylation sites, determination of the key sites responsible for toxicity is also imperative, along with clarification of their changes throughout the progression of disease. Moreover, determination of whether *O*-GlcNAc modifications influence protein aggregation, stability, or gene transcription to regulate neurotoxic effects or downstream signaling is of great importance. While thus study did not explicitly focus on aggregation of α-synuclein, investigation of the impact of *O*-GlcNAc cycling on the formation of neurofilament tangles that encompass various proteins including α-synuclein, is a promising avenue for exploration. Finally, unraveling the interplay between OGT and OGA in regulating *O*-GlcNAcylation during the course of PD presents another critical area for further research. These comprehensive studies should collectively contribute to a more in-depth understanding of the potential therapeutic implications of *O*-GlcNAcylation in PD.

## Materials and methods

### Cell culture

Mouse neuroblastoma cell line Neuro2a cells were obtained from the Korean Cell Line Bank (Seoul, Korea). Neuro2a is a mouse neural crest-derived cell line frequently utilized in Parkinson’s disease research [58]. They were cultured in Dulbecco’s modified Eagle’s medium (DMEM; HyClone, UT, USA) supplemented with 5% fetal bovine serum (FBS; HyClone) and an antibiotic mixture of penicillin and streptomycin (100 U/mL; HyClone). Regular testing for mycoplasma contamination was conducted on the cell lines to ensure their integrity and reliability for experimental use.

### Cell viability

Cell viability was assessed using a 3-(4,5-dimethylthiazol-2-yl)-5-(3-carboxy methoxyphenyl)-2-(4-sulfophenyl)-2H-tetrazolium inner salt (MTS) assay, which relies on a tetrazolium compound, MTS, and an electron acceptor agent, phenazine methosulfate (PMS, Promega Corp., WI, USA). In brief, cells were seeded in a 96-well plate at a density of 2 × 10^4^ cells per well in 200 μl medium, allowed to stabilize, and subsequently exposed to 6-OHDA (30 µM; Sigma-Aldrich, MO, USA) with or without GlcN (1 mM; Sigma-Aldrich). After 24 h, a mixture of 20 μl MTS/PMS solution was added to the culture medium with further incubation for 1–4 h at 37 °C. Absorbance was measured at 490 nm using a spectrophotometer.

### Western blotting

An equivalent quantity of total protein (30 μg) was separated using either 10% or 15% sodium dodecyl sulfate polyacrylamide gel electrophoresis (SDS-PAGE) and subsequently transferred to a nitrocellulose membrane (Hybond ECL; Amersham Pharmacia Biotech, NJ, USA). The membranes were blocked with 5% nonfat dried milk in phosphate-buffered saline (PBS) containing 0.05% Tween-20 (PBST) for 1 h at RT. After a washing with PBST, the blots were incubated overnight at 4 °C with specific antibodies: anti-*O*-GlcNAc (RL2, 1:1,000, sc-59623, Santa Cruz, CA, USA), anti-OGT (1:1,000, SC-74546, Santa Cruz, CA, USA), anti-OGA (1:1,000, 14711-1-AP, Proteintech, IL, USA), anti-TH (1:1,000, 2928, Invitrogen, CA, USA), anti-Bcl-xL (1:1,000, sc-56021Santa Cruz), anti-β-actin (1:1,000, sc-47778, Santa Cruz), anti-p-p65 (1:1,000, ab86299, Abcam, Cambrige, UK), anti-p65 (1:1,000, sc-8008, Santa Cruz), anti-p-IκB (1:1000, sc-101713, Santa Cruz), anti-IκB (1:1000, sc-371, Santa Cruz), anti-iNOS (1:1000, 610431, BD Bioscience, NJ, USA), anti-COX2 (1:1000, sc-19999, Santa Cruz), anti- IL-1β (1:1000, 12242, Cell Signaling Technology), anti-MFN2 (1:1000, 9482, Cell Signaling Technology, MA, USA), anti-OPA1 (1:1000, 80471, Cell Signaling Technology), anti-caspase 3 (1:1000, 9662, Cell Signaling Technology), and anti-cleaved caspase 3 (1:1000, 9664, Cell Signaling Technology). The blots were then incubated with horseradish peroxidase-conjugated anti-mouse or anti-rabbit antibodies (1:5,000, Santa Cruz). Immunoreactive proteins were subsequently detected using an enhanced chemiluminescence (ECL) western blotting detection system (Biomax, Kyunggi-Do, Korea).

### Immunocytochemistry and immunohistochemistry

Neuro2a cells or prepared brain sections were washed twice with PBST for 5 min at RT and then blocked with 0.5% bovine serum albumin in PBST. After blocking, the samples were incubated overnight at 4 °C with primary antibodies, including anti-TH (1:1,000, 2928, Invitrogen,), anti-OGA (1:100, 14711-1-AP, Proteintech), anti-OGT (1:100, sc-74546, Santa Cruz), anti-*O*-GlcNAc (1:100, sc-59623, Santa Cruz), anti-GFAP (1:100, G3893, Sigma-Aldrich), anti-Iba 1 (1:100, ab178864, Abcam), and anti-iNOS (1:100, 610431, BD Bioscience). Following the overnight incubation, the samples were washed three times with PBST (10 min per wash). Immunofluorescence labeling was carried out by incubating the cells with rabbit anti-IgG Alexa Fluor-488, 594 (Invitrogen) and mouse anti-IgG Alexa Fluor-488, 594 (Invitrogen). ROS was measured by DCFDA (Sigma-Aldrich).The cell nuclei were counterstained with 4,6-diamidino-2-phenylindole (DAPI, Invitrogen). Immunohistochemical staining for iNOS and TH was visualized using a 0.05% DAB staining method (Vector Laboratories, CA, USA). The immunofluorescence analysis was conducted using a Zeiss LSM 500 confocal imaging system (Zeiss, Oberkochen, Germany). The DAB-stained samples were examined using bright-field microscopy (Olympus, Tokyo, Japan).

### Stereological counting of TH-positive neuron

Immunostained TH neurons in the SN on one hemisphere of the stereotactic brain were counted across the entire extent of the SN [59]. To ensure accuracy, TH-positive neurons were only counted when the nucleus was optimally visualized, which occurred in a single focal plane. The average cell count of three mice in each experimental group was plotted on a graph.

### RNA extraction and real-time polymerase chain reaction (RT-PCR)

Total RNA was isolated from the cells using TRIzol™ (Invitrogen, CA, USA) according to the manufacturer’s instructions. Complementary DNA (cDNA) was generated from 1 μg of RNA using GoScript Reverse Transcriptase (Promega) following the manufacturer’s instructions. Subsequently, PCR was carried out utilizing mouse-specific primers for each target (MFN1, F: CCGGAGTACATGGAGGATGTG, R: GAAATCCTTCTGCAAGTGCCC; MFN2, F: GCCAGTTTGTGGAATACGCC, R: AGTGAATCCAGAGCCTCGAC; OPA1, F: ACCTTGCCAGTTTAGCTCCC, R: ACCTTCCTGTAATGCTTGTCACT, FIS1, F: AGAGACGAAGCTGCAAGGAA, R: CATAGTCCCGCTGTTCCTCTT; TFAM, F: GGCAAAGGATGATTCGGCTC, R: GATCGTTTCACACTTCGACGG; GAPDH, F: AATGTGTCCGTCGTGGATCT, R: AAGTCGCAGGAGAGACAACCTG). The cDNAs were amplified using SYBR Green Real-time PCR Master Mix (BIOFACT, Daejeon, Korea). Expression levels were normalized to the expression of GAPDH.

### Nitrite assay

Nitric oxide (NO) production was assessed by quantifying the nitrite levels in the culture medium, following a procedure described previously [60] with minor adjustments. In summary, 50 μl of the culture medium was mixed with 50 μl of Griess reagent (composed of 1% sulfanilamide, 0.1% naphthylenediamine, and 5% phosphoric acid). The optical density at 540 nm (OD 540) was determined using a microplate reader (Bio-Tek Instruments, TX, USA). Nitrite concentrations were determined by comparing the optical density to a standard solution of NaN2.

### Electron microscopy

For electron microscopy (EM) analysis, Neuro2a cells or brain tissues were fixed with a solution containing 2% glutaraldehyde and paraformaldehyde in 0.1 M phosphate buffer (pH 7.4) for 12 h. They were then rinsed twice for 30 min in 0.1 M phosphate buffer. Subsequently, the specimens were post-fixed with 1% osmium tetroxide (OsO4) dissolved in 0.1 M phosphate buffer for 2 h, dehydrated using a graded series of ethanol (ranging from 50% to 100%), and then infiltrated with propylene oxide. The specimens were embedded using a Poly/Bed 812 kit (Polysciences, PA, USA). After embedding in fresh, pure resin and polymerization at 65 °C in an electron microscope oven (TD-700, DOSAKA, Koyto, Japan) for 24 h, thick sections (200 nm) were initially cut and stained with toluidine blue for light microscopy. Additionally, thin sections (80 nm) were prepared and double-stained with 3% uranyl acetate and lead citrate for contrast staining. The sections were cut using a Leica EM UC7 Ultra-microtome (Leica Microsystems, IL, USA). All thin sections were observed using transmission electron microscopy (JEM-1011, JEOL, MA, USA) at an acceleration voltage of 80 kV. The percentage of damaged mitochondria was calculated.

### Measurement of mitochondrial oxygen consumption

Mitochondrial oxygen consumption was assessed using polarographic high-resolution respirometry employing the Oxygraph-2k system (OROBOROS, Innsbruck, Austria) at a temperature of 30 °C in assay buffer (buffer Z containing 50 μM EGTA and 20 mM creatine). Oxygen consumption was measured under three different assay conditions: (a) 5 mM glutamate (a substrate of complex I in the electron transport chain (ETC) in mitochondria) + 2 mM malate (another substrate of complex I), (b) 4 mM ADP (a substrate for ATP production in mitochondria), and (c) 10 mM succinate (a substrate of complex II in the ETC). Upon completion of the experiment, mitochondrial O_2_ respiration was normalized based on the cell counts of each sample.

### Mitotracker-Red staining

Cells were seeded on 16 mm glass coverslips and incubated with MitoTracker Red (Thermo Fisher Scientific, MA, USA) at a final concentration of 1 μM for 15 min at 37 °C under 5% CO_2_. Subsequently, cells were rinsed once with PBS for paraformaldehyde fixation, followed by another wash before being mounted onto glass slides using Dako fluorescent mounting medium (Sigma-Aldrich).

### Experimental mice and drug administration

This study was conducted with approval from the Institutional Animal Care and Use Committee of Inha University in Incheon, Korea (Approval Number: 190920-665). Male C57BL/6 J mice (7 weeks old; DBL, Chungbuk, Korea) were acclimated in a controlled environment under a 12 h light/dark cycle for 1 week before the commencement of drug administration. Targeted injection of 6-OHDA (5 μg/2 μl) into the striatum (ST) of mouse brain was conducted using specific coordinates (0.4 mm posterior to bregma, ± 2.2 mm lateral to midline and –3.5 mm ventral to the brain surface) with a stainless steel (26-gauge) injection needle connected to a 10 μl microsyringe (Hamilton, TX, USA). The needle was left in place for 10 min before being slowly withdrawn. Glucosamine (20 mg/kg) was administered intraperitoneally three times a week for 4 weeks. The sample size of each group in this study have been determined based on previous publication (Hwang et al., 2010), practical considerations, and ethical guideline. Power analysis was performed to ensure the study had sufficient statistical power to detect significant effects confidently. Mice showing signs of distress or abnormal behavior during the acclimation period were excluded to reduce the risk of bias in the results. The randomization process for allocating experimental groups was carried out by an individual who was not directly involved in the experimental procedures to uphold objectivity and integrity. Blinding was ensured by assigning separate personnel responsible for group allocation and outcome assessment.

### Virus administration in animals through stereotaxic surgery

The adeno-associated virus serotype 8 (AAV8) vector containing mouse OGT (mOGT), AAV8-CMV-mOGT-Myc-Flag (AAV-OGT), was generated and purified by the Korea Institute of Science and Technology (KIST) Virus Facility (KIST, Seoul, Korea). AAV8 was engineered using DNA family shuffling technology, resulting in a hybrid capsid derived from eight standard AAV serotypes. The plasmids used for AAV vector production included the AAV8 serotype construct, the AAV transfer plasmid, a plasmid encoding rep and serotype-specific capsid proteins, and a plasmid encoding adenoviral helper sequences. The viral vectors were pseudotyped, with mOGT flanked by inverted terminal repeats of AAV8, and encapsulated within an AAV8 capsid. The mice were randomly allocated to one of three groups: vector-only (control), vector-only with 6-OHDA, and 6-OHDA with AAV-OGT. During the injection, mice received 4 μl of either the pAAV-CMV control virus or AAV8-OGT virus. Before the injection, the mice were anesthetized using isoflurane (Baxter, IL, USA). The animals were then administered 4 μl of AAV-OGT (~1 × 10^13^ genome copies per milliliter) into the right and left substantia nigra at a flow rate of 0.3 μl/min.

### Behavioral test

#### Pole test

Each mouse was positioned atop a vertical wooden pole with a rough surface, measuring 1 cm in diameter and 50 cm in height. A day before testing, the mice were acclimated to the apparatus by being placed on top of the pole and allowed to descend five times. The total duration required for each mouse to reach the base of the pole and place all four paws on the floor was meticulously recorded. In instances where a mouse was unable to complete the descent, fell off, or slipped down the pole, a default value of 120 s was assigned.

#### Cylinder test

Mouse was placed in a glass cylinder and observed for a duration of 5–10 min. Forelimb asymmetry was evaluated by scoring independent, weight-bearing contacts on the cylinder wall made by either the ipsilateral or contralateral paw, relative to the lesioned hemisphere. This assessment also took into account movements made by both paws. The percentage of touches on the ipsilateral and contralateral sides, relative to the total number of touches (ipsilateral + contralateral + both = total), was calculated.

#### Apomorphine rotation test

Each mouse was situated in a mouse cage and permitted to acclimate to the environment for a period of 10 min before the rotation test. Subsequently, mice received an intraperitoneal injection of 0.5 mg/kg apomorphine and were then returned to their cages. Full 360° turns in the contralateral direction to the lesion were manually counted over a 30 min interval, using a stopwatch. The collected data were subjected to analysis in order to determine the number of contralateral rotations.

### Statistical analyses

The mean differences between experimental groups were assessed using either a Student *t*-test or a one-way analysis of variance (ANOVA), followed by a Tukey’s post-hoc test. Statistically significant differences were defined as those with a *p*-value <0.05.

### Supplementary information

Suppl. Figure legends

Suppl. Fuigure 1

Suppl. Figure 2

checklist

Uncropped Western Blot

## Data Availability

The data and materials used in this research are available upon request from the corresponding author.

## References

[1] Damier P, Hirsch EC, Agid Y, Graybiel AM (1999). The substantia nigra of the human brain. II. Patterns of loss of dopamine-containing neurons in Parkinson’s disease. Brain.

[2] Dauer W, Przedborski S (2003). Parkinson’s disease: mechanisms and models. Neuron.

[3] Jankovic J, Stacy M (2007). Medical management of levodopa-associated motor complications in patients with Parkinson’s disease. CNS Drugs.

[4] Bové J, Perier C (2012). Neurotoxin-based models of Parkinson’s disease. Neuroscience.

[5] Blandini F, Armentero MT (2012). Animal models of Parkinson’s disease. Febs j.

[6] Blandini F, Levandis G, Bazzini E, Nappi G, Armentero MT (2007). Time-course of nigrostriatal damage, basal ganglia metabolic changes and behavioural alterations following intrastriatal injection of 6-hydroxydopamine in the rat: new clues from an old model. Eur J Neurosci.

[7] Spieles-Engemann AL, Collier TJ, Sortwell CE (2010). A functionally relevant and long-term model of deep brain stimulation of the rat subthalamic nucleus: advantages and considerations. Eur J Neurosci.

[8] Carvalho MM, Campos FL, Coimbra B, Pêgo JM, Rodrigues C, Lima R (2013). Behavioral characterization of the 6-hydroxidopamine model of Parkinson’s disease and pharmacological rescuing of non-motor deficits. Mol Neurodegener.

[9] Cenci MA, Lee CS, Björklund A (1998). L-DOPA-induced dyskinesia in the rat is associated with striatal overexpression of prodynorphin- and glutamic acid decarboxylase mRNA. Eur J Neurosci.

[10] Cenci, MA, M Lundblad. Ratings of L-DOPA-induced dyskinesia in the unilateral 6-OHDA lesion model of Parkinson’s disease in rats and mice. Curr Protoc Neurosci. 2007;9:Unit 9.25. 10.1002/0471142301.ns0925s41. 10.1002/0471142301.ns0925s41 18428668

[11] Glinka YY, Youdim MB (1995). Inhibition of mitochondrial complexes I and IV by 6-hydroxydopamine. Eur J Pharm.

[12] Afshin-Majd S, Bashiri K, Kiasalari Z, Baluchnejadmojarad T, Sedaghat R, Roghani M (2017). Acetyl-l-carnitine protects dopaminergic nigrostriatal pathway in 6-hydroxydopamine-induced model of Parkinson’s disease in the rat. Biomed Pharmacother.

[13] Burton A (2006). mtDNA deletions associated with ageing and PD. Lancet Neurol.

[14] Ni HM, Williams JA, Ding WX (2015). Mitochondrial dynamics and mitochondrial quality control. Redox Biol.

[15] Chen W, Zhao H, Li Y (2023). Mitochondrial dynamics in health and disease: mechanisms and potential targets. Signal Transduct Target Ther.

[16] Aiken J, Holzbaur ELF (2021). Cytoskeletal regulation guides neuronal trafficking to effectively supply the synapse. Curr Biol.

[17] Bose A, Beal MF (2016). Mitochondrial dysfunction in Parkinson’s disease. J Neurochem.

[18] McGeer PL, Itagaki S, Boyes BE, McGeer EG (1988). Reactive microglia are positive for HLA-DR in the substantia nigra of Parkinson’s and Alzheimer’s disease brains. Neurology.

[19] Taylor JM, Main BS, Crack PJ (2013). Neuroinflammation and oxidative stress: co-conspirators in the pathology of Parkinson’s disease. Neurochem Int.

[20] Duke DC, Moran LB, Pearce RK, Graeber MB (2007). The medial and lateral substantia nigra in Parkinson’s disease: mRNA profiles associated with higher brain tissue vulnerability. Neurogenetics.

[21] Lin JC, Lin CS, Hsu CW, Lin CL, Kao CH (2016). Association between Parkinson’s disease and inflammatory bowel disease: a nationwide taiwanese retrospective cohort study. Inflamm Bowel Dis.

[22] Mosley RL, Hutter-Saunders JA, Stone DK, Gendelman HE (2012). Inflammation and adaptive immunity in Parkinson’s disease. Cold Spring Harb Perspect Med.

[23] Stephen HM, Adams TM, Wells L (2021). Regulating the regulators: mechanisms of substrate selection of the O-GlcNAc cycling enzymes OGT and OGA. Glycobiology.

[24] Zhu Y, Shan X, Yuzwa SA, Vocadlo DJ (2014). The emerging link between O-GlcNAc and Alzheimer disease. J Biol Chem.

[25] Yuzwa SA, Shan X, Macauley MS, Clark T, Skorobogatko Y, Vosseller K (2012). Increasing O-GlcNAc slows neurodegeneration and stabilizes tau against aggregation. Nat Chem Biol.

[26] Kim C, Nam DW, Park SY, Song H, Hong HS, Boo JH (2013). O-linked β-N-acetylglucosaminidase inhibitor attenuates β-amyloid plaque and rescues memory impairment. Neurobiol Aging.

[27] Balana AT, Mahul-Mellier AL, Nguyen BA, Horvath M, Javed A, Hard ER, et al. O-GlcNAc modification forces the formation of an α-Synuclein amyloid-strain with notably diminished seeding activity and pathology. bioRxiv. 2023. 10.1101/2023.03.07.53157.

[28] Wu K, Li D, Xiu P, Ji B, Diao J (2020). O-GlcNAcylation inhibits the oligomerization of alpha-synuclein by declining intermolecular hydrogen bonds through a steric effect. Phys Biol.

[29] Galesic A, Pratt MR (2020). Investigating the effects of O-GlcNAc modifications in Parkinson’s disease using semisynthetic α-synuclein. Methods Mol Biol.

[30] Levine PM, Galesic A, Balana AT, Mahul-Mellier AL, Navarro MX, De Leon CA (2019). α-Synuclein O-GlcNAcylation alters aggregation and toxicity, revealing certain residues as potential inhibitors of Parkinson’s disease. Proc Natl Acad Sci USA.

[31] Eldeeb MA, Thomas RA, Ragheb MA, Fallahi A, Fon EA (2022). Mitochondrial quality control in health and in Parkinson’s disease. Physiol Rev.

[32] Blesa J, Przedborski S (2014). Parkinson’s disease: animal models and dopaminergic cell vulnerability. Front Neuroanat.

[33] Slawson C, Zachara NE, Vosseller K, Cheung WD, Lane MD, Hart GW (2005). Perturbations in O-linked beta-N-acetylglucosamine protein modification cause severe defects in mitotic progression and cytokinesis. J Biol Chem.

[34] Park J, Lee Y, Jung EH, Kim SM, Cho H, Han IO (2020). Glucosamine regulates hepatic lipid accumulation by sensing glucose levels or feeding states of normal and excess. Biochim Biophys Acta Mol Cell Biol Lipids.

[35] Decourcelle A, Loison I, Baldini S, Leprince D, Dehennaut V (2020). Evidence of a compensatory regulation of colonic O-GlcNAc transferase and O-GlcNAcase expression in response to disruption of O-GlcNAc homeostasis. Biochem Biophys Res Commun.

[36] Ortiz-Meoz RF, Jiang J, Lazarus MB, Orman M, Janetzko J, Fan C (2015). A small molecule that inhibits OGT activity in cells. ACS Chem Biol.

[37] Burén S, Gomes AL, Teijeiro A, Fawal MA, Yilmaz M, Tummala KS (2016). Regulation of OGT by URI in response to glucose confers c-MYC-dependent survival mechanisms. Cancer Cell.

[38] Kazemi Z, Chang H, Haserodt S, McKen C, Zachara NE (2010). O-linked beta-N-acetylglucosamine (O-GlcNAc) regulates stress-induced heat shock protein expression in a GSK-3beta-dependent manner. J Biol Chem.

[39] Zachara NE, O'Donnell N, Cheung WD, Mercer JJ, Marth JD, Hart GW (2004). Dynamic O-GlcNAc modification of nucleocytoplasmic proteins in response to stress. A survival response of mammalian cells. J Biol Chem.

[40] Zhao L, Feng Z, Yang X, Liu J (2016). The regulatory roles of O-GlcNAcylation in mitochondrial homeostasis and metabolic syndrome. Free Radic Res.

[41] Malpartida AB, Williamson M, Narendra DP, Wade-Martins R, Ryan BJ (2021). Mitochondrial dysfunction and mitophagy in Parkinson’s disease: from mechanism to therapy. Trends Biochem Sci.

[42] Pickrell AM, Youle RJ (2015). The roles of PINK1, parkin, and mitochondrial fidelity in Parkinson’s disease. Neuron.

[43] Tan EP, Villar MT, E L, Lu J, Selfridge JE, Artigues A (2014). Altering O-linked β-N-acetylglucosamine cycling disrupts mitochondrial function. J Biol Chem.

[44] Tan EP, McGreal SR, Graw S, Tessman R, Koppel SJ, Dhakal P (2017). Sustained O-GlcNAcylation reprograms mitochondrial function to regulate energy metabolism. J Biol Chem.

[45] Weimer S, Priebs J, Kuhlow D, Groth M, Priebe S, Mansfeld J (2014). D-glucosamine supplementation extends life span of nematodes and of ageing mice. Nat Commun.

[46] Macchi B, Di Paola R, Marino-Merlo F, Felice MR, Cuzzocrea S, Mastino A (2015). Inflammatory and cell death pathways in brain and peripheral blood in Parkinson’s disease. CNS Neurol Disord Drug Targets.

[47] Imamura K, Hishikawa N, Sawada M, Nagatsu T, Yoshida M, Hashizume Y (2003). Distribution of major histocompatibility complex class II-positive microglia and cytokine profile of Parkinson’s disease brains. Acta Neuropathol.

[48] Glass CK, Saijo K, Winner B, Marchetto MC, Gage FH (2010). Mechanisms underlying inflammation in neurodegeneration. Cell.

[49] Konnova, EA, M Swanberg. Animal models of Parkinson’s disease. In: TB Stoker and JC Greenland, editors. Parkinson’s disease: pathogenesis and clinical aspects, Brisbane (AU) Codon Publications; 2018;5.

[50] Singh SS, Rai SN, Birla H, Zahra W, Rathore AS, Singh SP (2020). NF-κB-mediated neuroinflammation in Parkinson’s disease and potential therapeutic effect of polyphenols. Neurotox Res.

[51] Ghosh A, Roy A, Liu X, Kordower JH, Mufson EJ, Hartley DM (2007). Selective inhibition of NF-kappaB activation prevents dopaminergic neuronal loss in a mouse model of Parkinson’s disease. Proc Natl Acad Sci USA.

[52] Flood PM, Qian L, Peterson LJ, Zhang F, Shi JS, Gao HM (2011). Transcriptional Factor NF-κB as a target for therapy in Parkinson’s disease. Parkinsons Dis.

[53] Hwang SY, Hwang JS, Kim SY, Han IO (2013). Glucosamine inhibits lipopolysaccharide-stimulated inducible nitric oxide synthase induction by inhibiting expression of NF-kappaB/Rel proteins at the mRNA and protein levels. Nitric Oxide.

[54] Hwang SY, Shin JH, Hwang JS, Kim SY, Shin JA, Oh ES (2010). Glucosamine exerts a neuroprotective effect via suppression of inflammation in rat brain ischemia/reperfusion injury. Glia.

[55] Lee Y, Lee S, Park JW, Hwang JS, Kim SM, Lyoo IK (2018). Hypoxia-induced neuroinflammation and learning-memory impairments in adult zebrafish are suppressed by glucosamine. Mol Neurobiol.

[56] Park J, Jung S, Kim SM, Park IY, Bui NA, Hwang GS (2021). Repeated hypoxia exposure induces cognitive dysfunction, brain inflammation, and amyloidβ/p-Tau accumulation through reduced brain O-GlcNAcylation in zebrafish. J Cereb Blood Flow Metab.

[57] Fujita KA, Ostaszewski M, Matsuoka Y, Ghosh S, Glaab E, Trefois C (2014). Integrating pathways of Parkinson’s disease in a molecular interaction map. Mol Neurobiol.

[58] Delaidelli A, Richner M, Jiang L, van der Laan A, Bergholdt Jul Christiansen I, Ferreira N (2021). α-synuclein pathology in Parkinson disease activates homeostatic NRF2 anti-oxidant response. Acta Neuropathol Commun.

[59] Oh SH, Kim HN, Park HJ, Shin JY, Kim DY, Lee PH (2017). The cleavage effect of mesenchymal stem cell and its derived matrix metalloproteinase-2 on extracellular α-synuclein aggregates in Parkinsonian models. Stem Cells Transl Med.

[60] Hwang JS, Jung EH, Kwon MY, Han IO (2016). Glioma-secreted soluble factors stimulate microglial activation: The role of interleukin-1β and tumor necrosis factor-α. J Neuroimmunol.

