# Supplementary material for: Protective effect of increased O-GlcNAc cycling against 6-OHDA induced Parkinson’s disease pathology
Source: Cell Death Dis. 2024 Apr 23;15(4):287. doi: 10.1038/s41419-024-06670-1 (PMC11039476; doi:10.1038/s41419-024-06670-1)
Supplement: Supplementary file 1 — Suppl. Figure legends [file 41419_2024_6670_MOESM1_ESM.docx]

**Supplementary figure 1. Regulation of *O*-GlcNAc by 6-OHDA in Neuro2a cells**

Neuro2a cells were treated with 30μM 6-OHDA for 3, 6, 12, and 24 hours (A), or various concentrations (1μM, 5μM, 10μM, and 30μM) of 6-OHDA for 24 hours. Representative immunofluorescence images (20X) for *O*-GlcNAc (red), DAPI (blue) and merged images are shown. (n=3/group). The scale bar represents 50 μm. Data is presented as mean ± SEM; ^*^*p*<0.05, ^***^*p*<0.001. Statistical analysis was performed using one-way ANOVA with Tukey's post hoc multiple comparison test.

**Supplementary figure 2. Effect of GlcN on 6-OHDA-induced *O*-GlcNAc changes in Neuro2a cells**

Neuro2a cells were treated with 30μM of 6-OHDA, either with or without 1mM of GlcN, for 24 hours. Representative immunofluorescence images (20X) of *O*-GlcNAc (green), DAPI (blue), and merged images are displayed. (n=3/group). The scale bar represents 50 μm. Data is presented as mean ± SEM; ^*^*p*<0.05. Statistical analysis was conducted using one-way ANOVA with Tukey's post hoc multiple comparison test.
