# Supplementary figures and images for: Protective effect of increased O-GlcNAc cycling against 6-OHDA induced Parkinson’s disease pathology
Source: Cell Death Dis. 2024 Apr 23;15(4):287. doi: 10.1038/s41419-024-06670-1 (PMC11039476; doi:10.1038/s41419-024-06670-1)

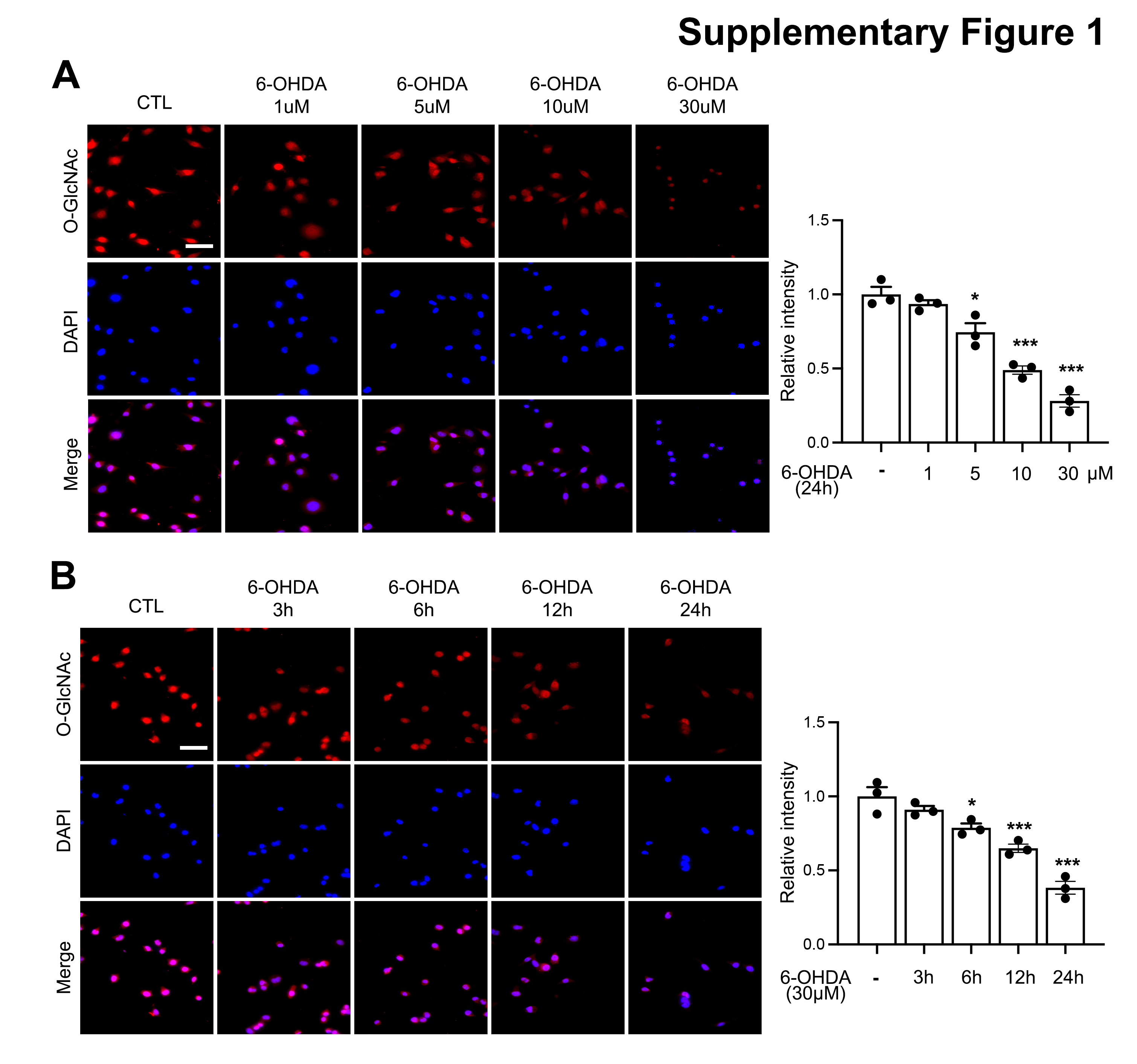

Supplement: Supplementary file 2 — Suppl. Fuigure 1 [file 41419_2024_6670_MOESM2_ESM.tif]

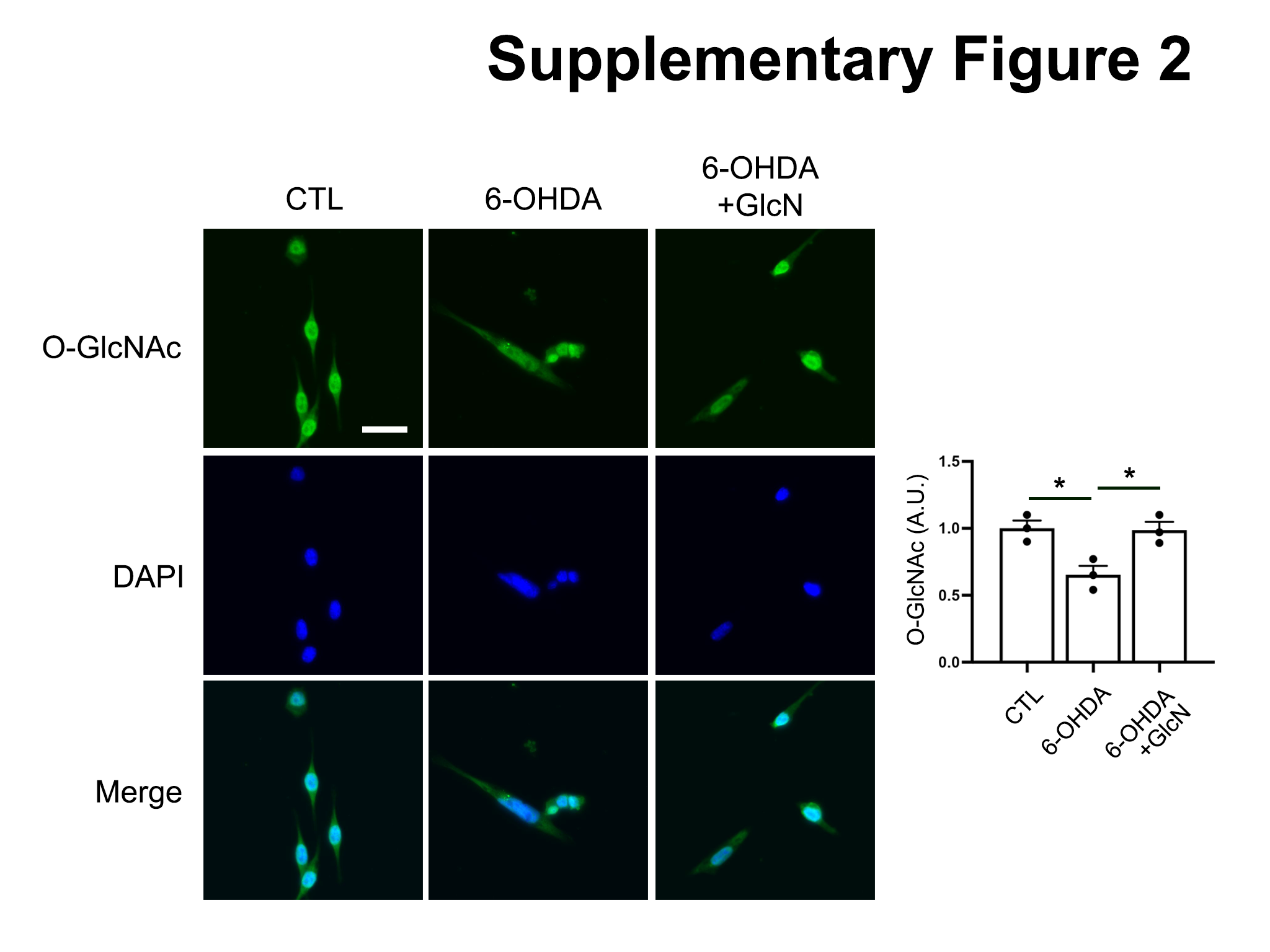

Supplement: Supplementary file 3 — Suppl. Figure 2 [file 41419_2024_6670_MOESM3_ESM.tif]

Figure 1

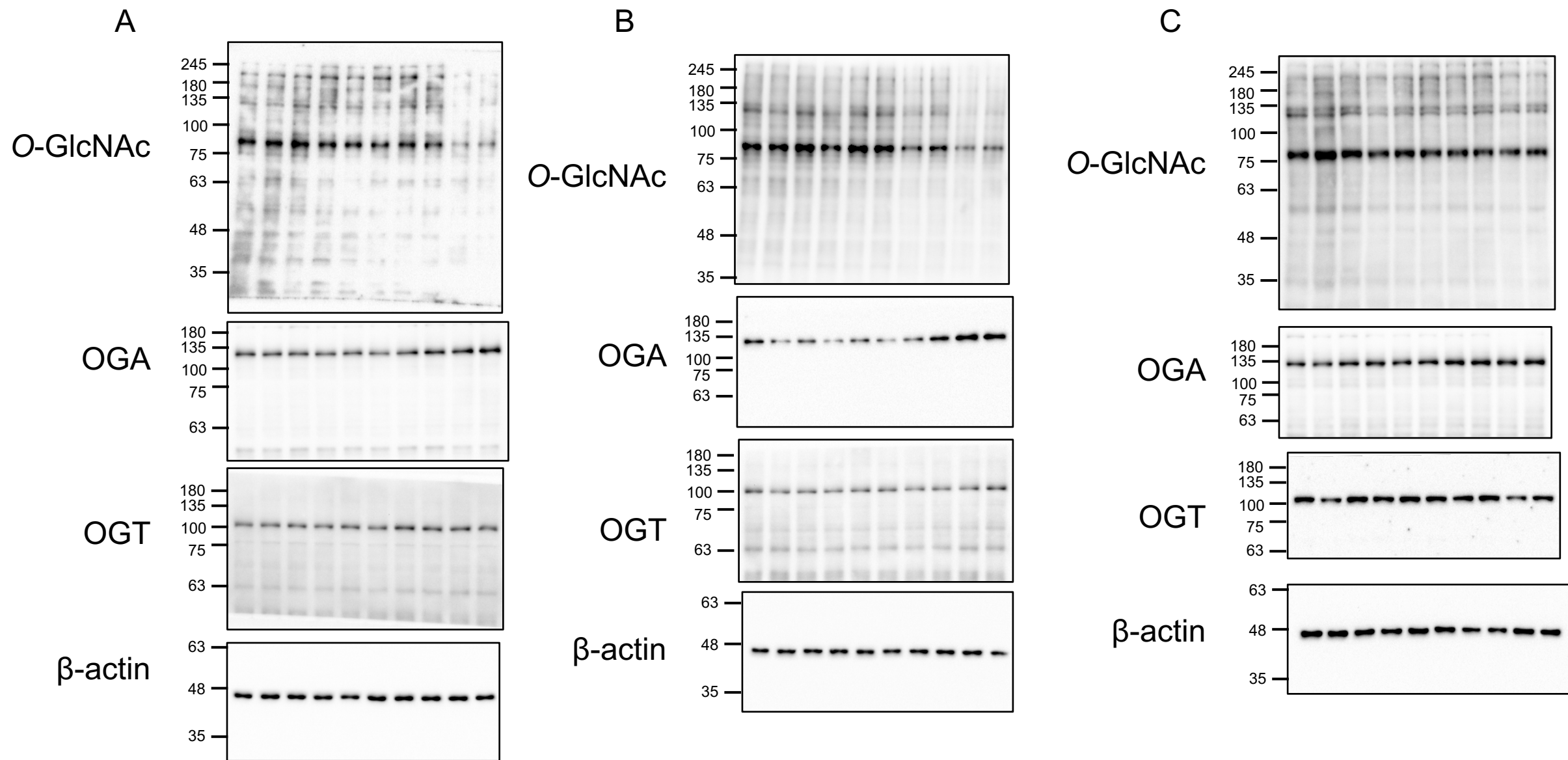

Figure 2

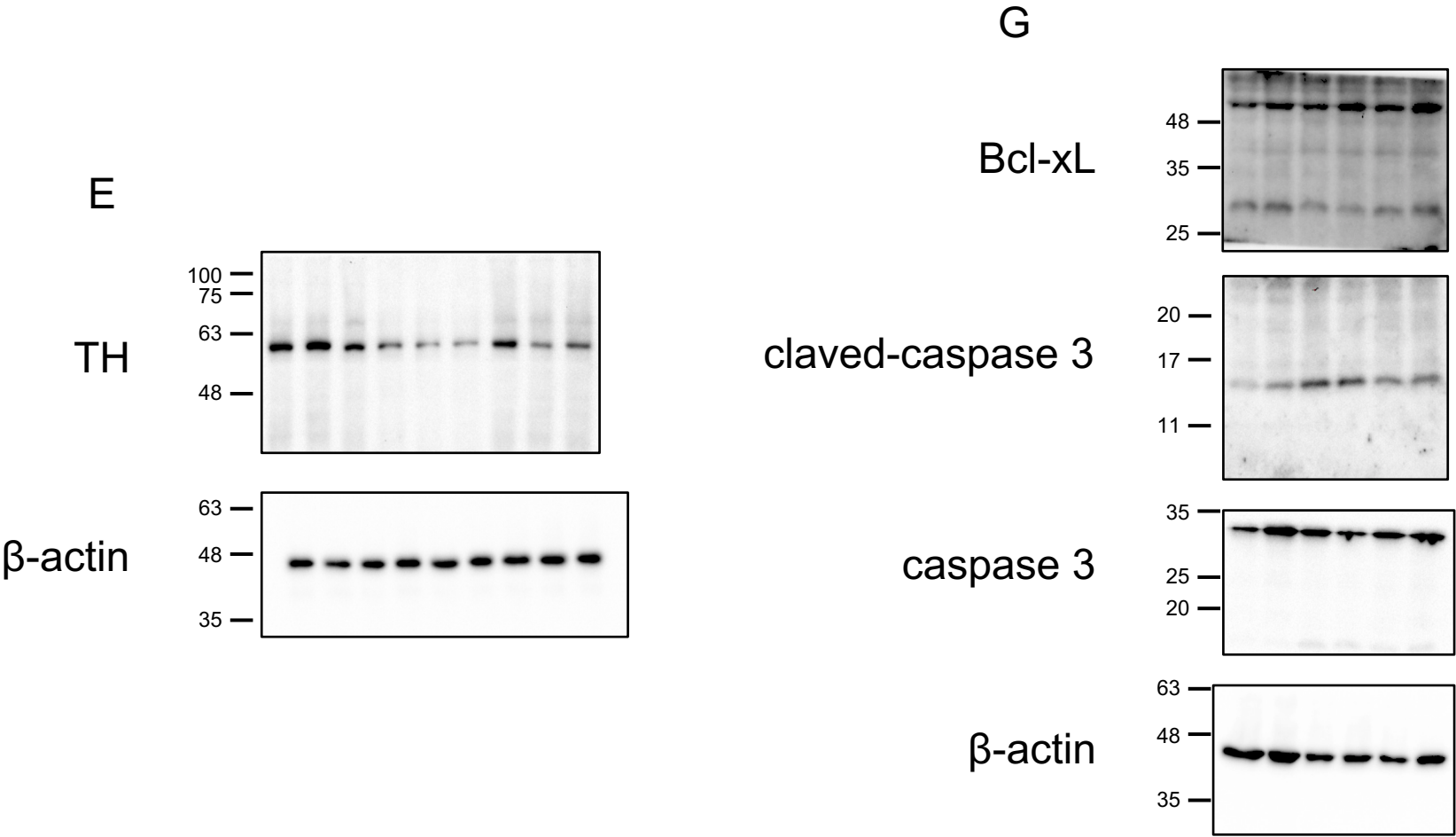

Figure 3

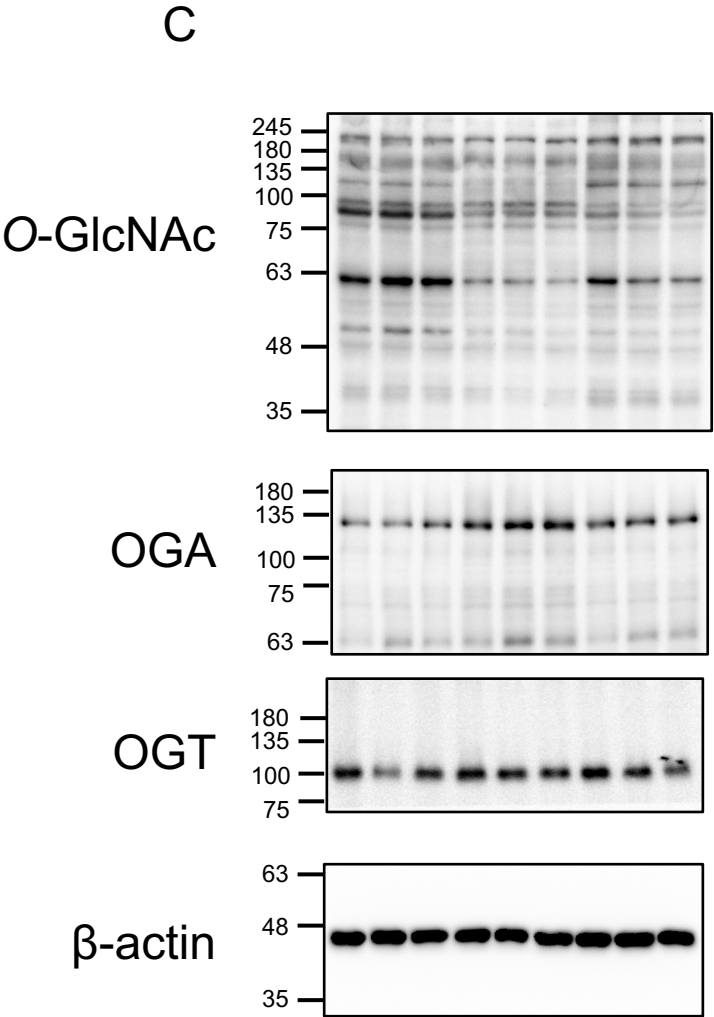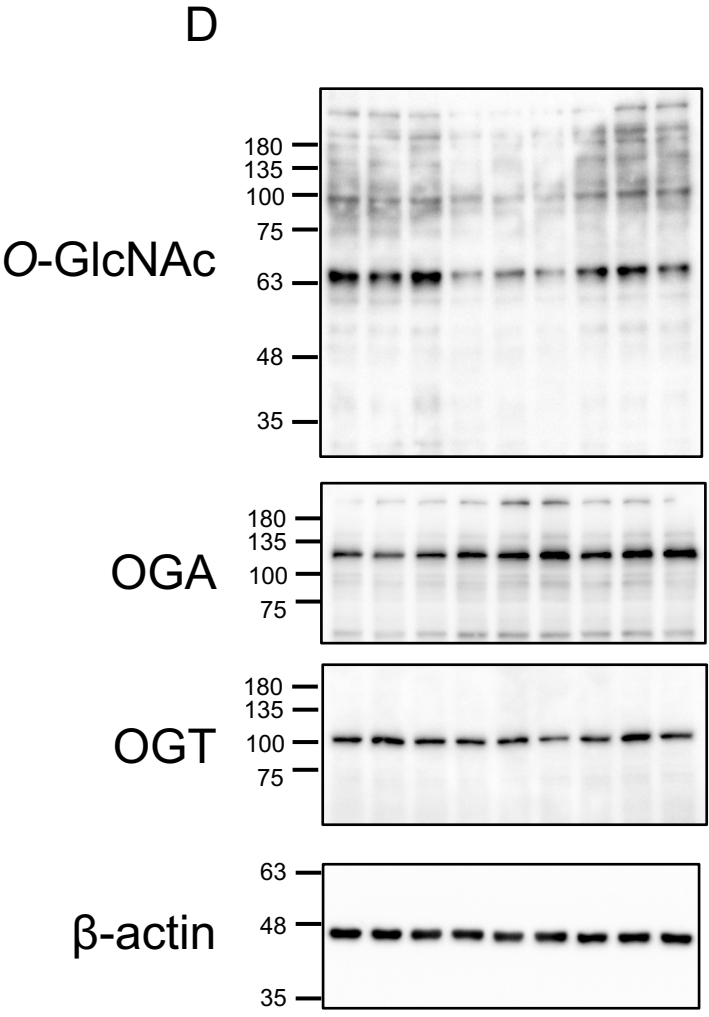

Figure 4

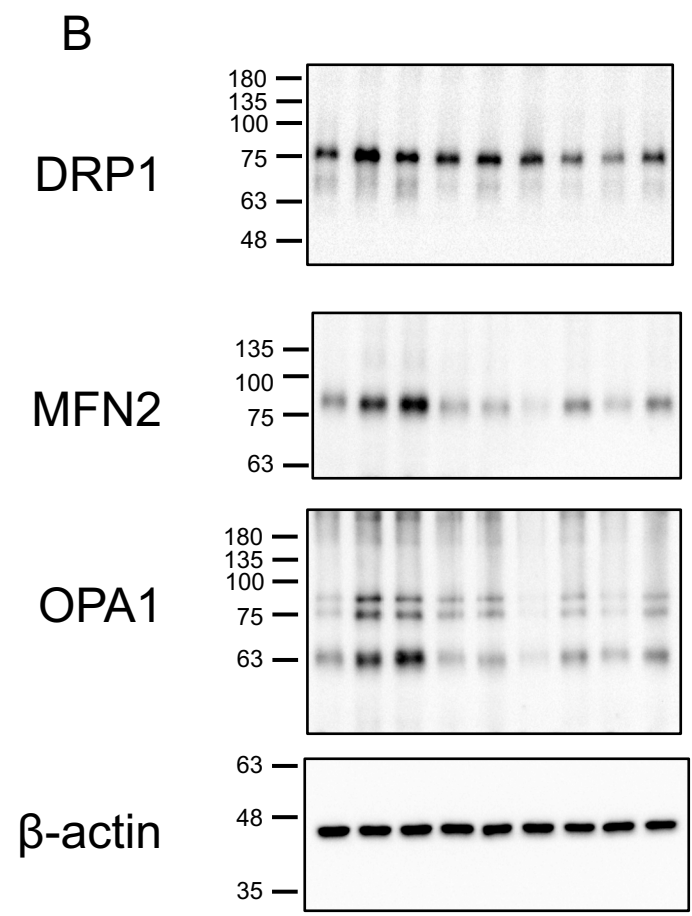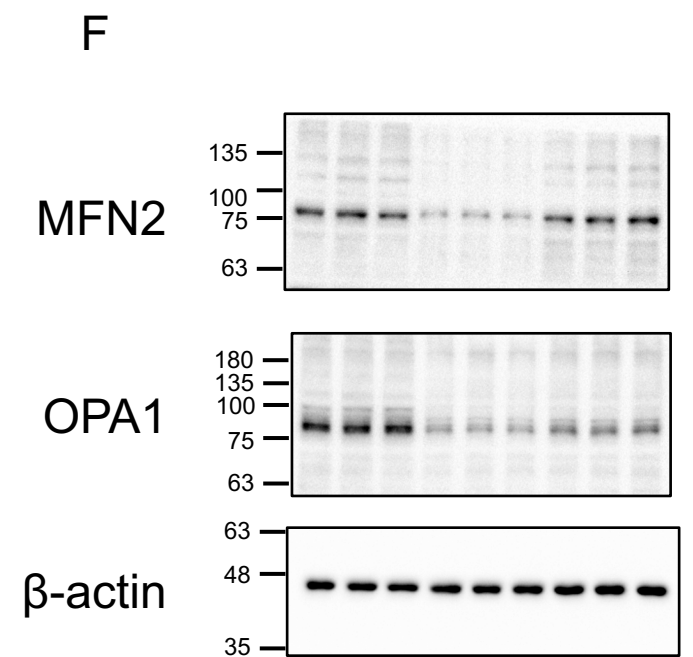

Figure 5

C

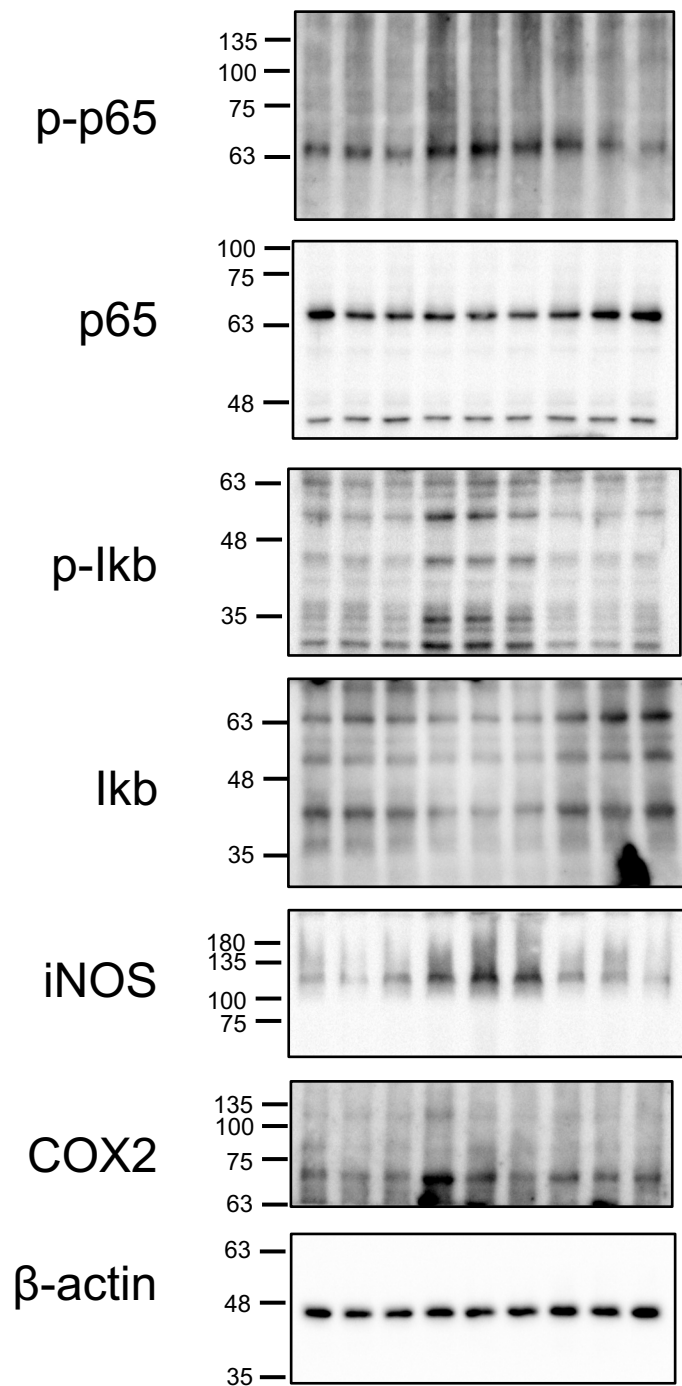

E

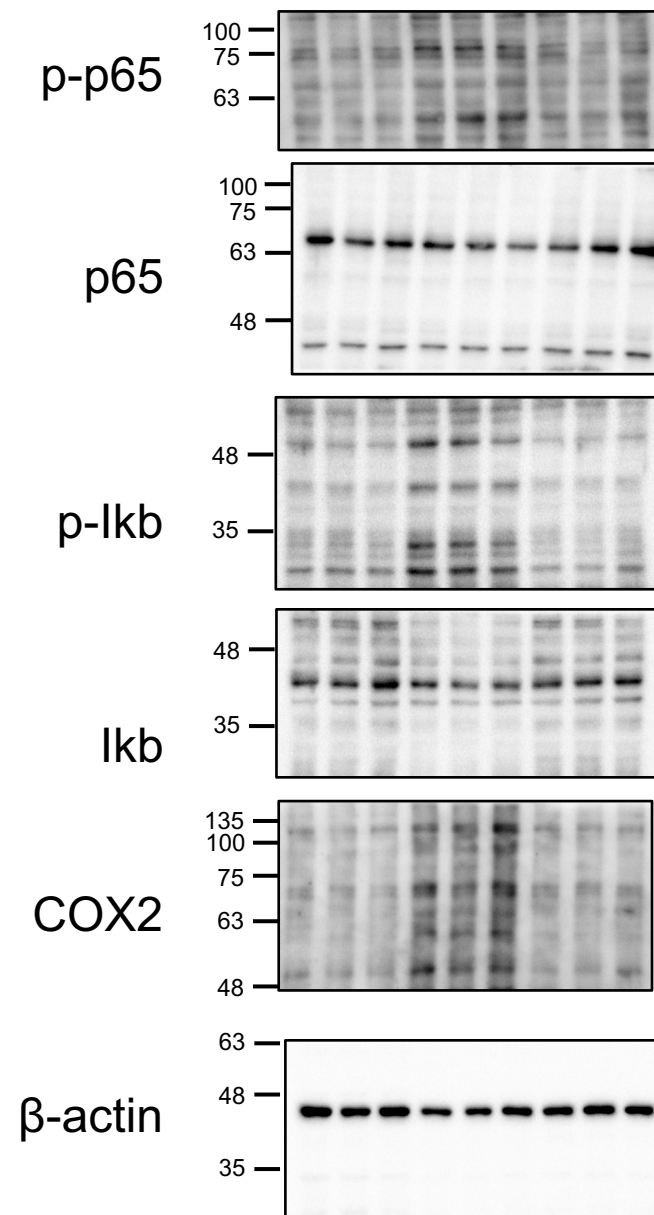

Figure 6

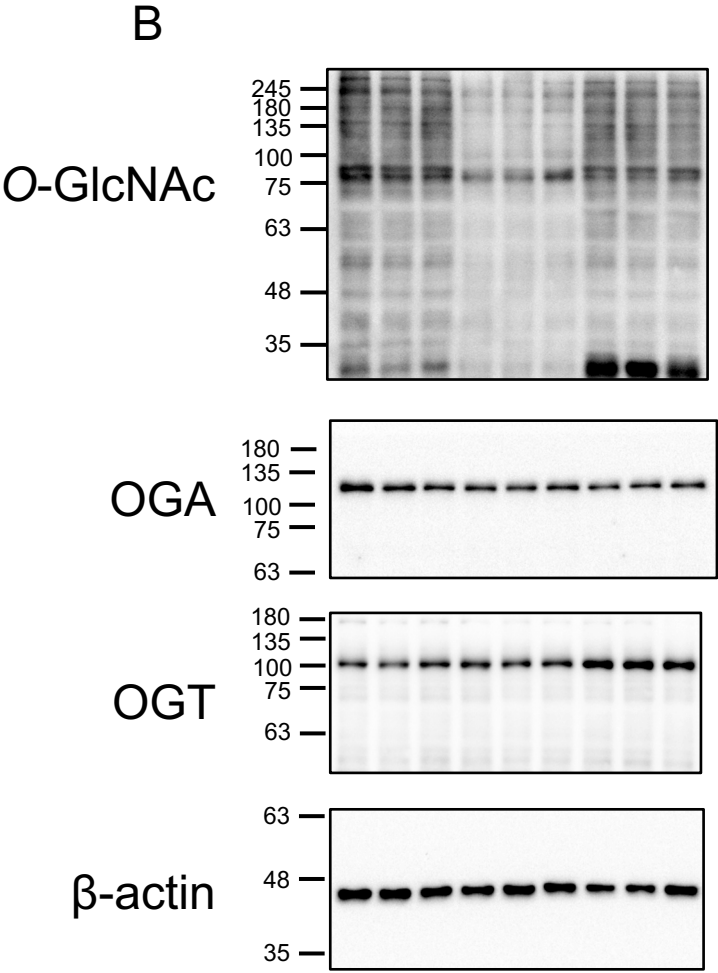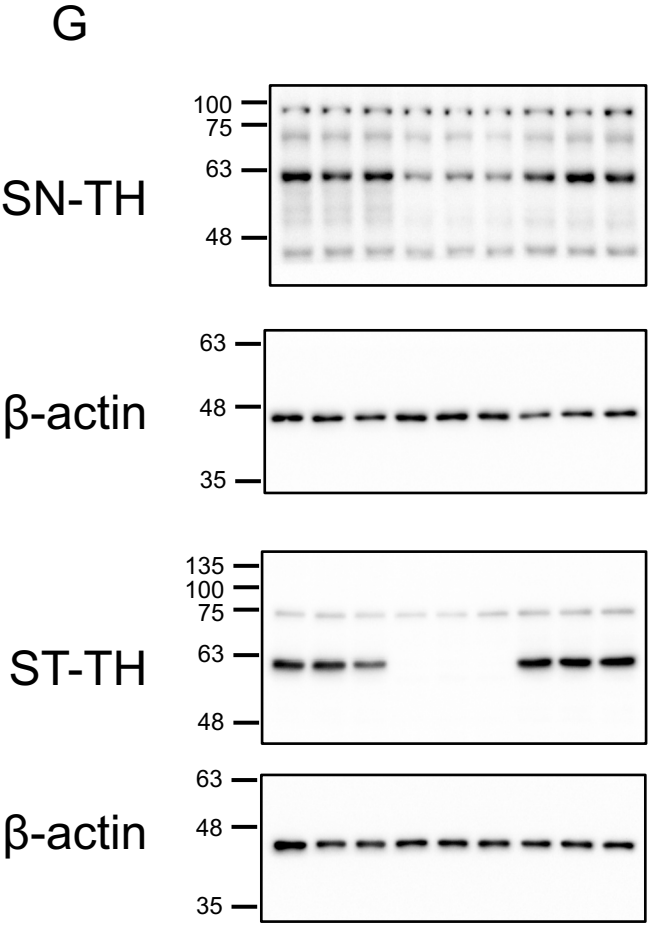

Figure 7

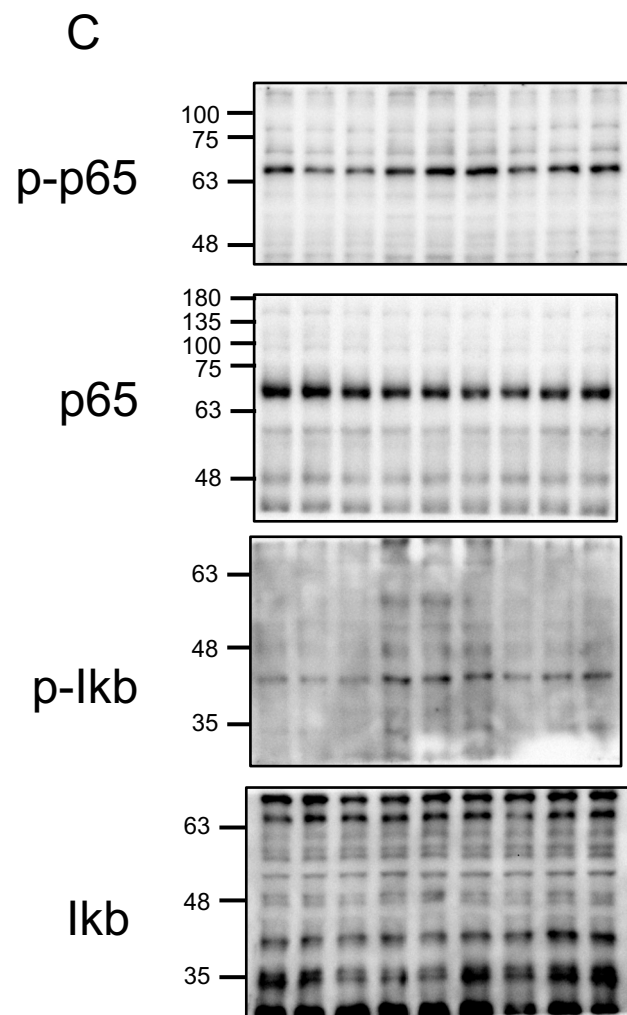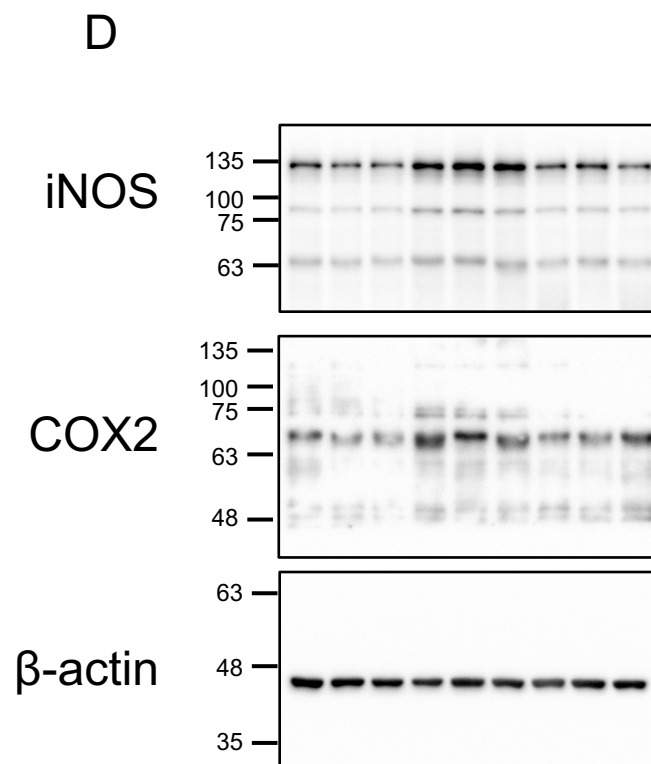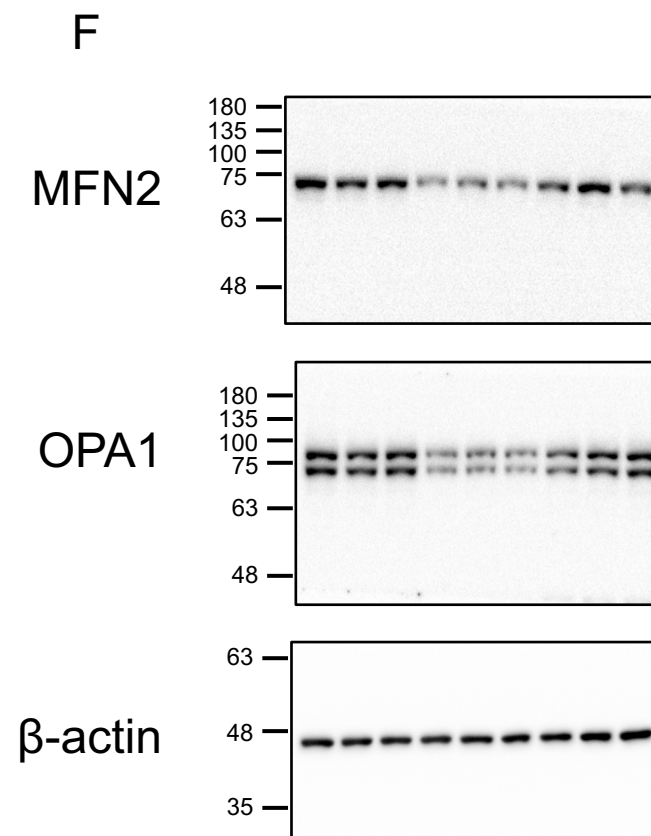

Supplement: Supplementary file 5 — Uncropped Western Blot [file 41419_2024_6670_MOESM5_ESM.pdf]
